# Supplementary figures and images for: Critical role of P-Glycoprotein-9 in ivermectin tolerance in nematodes
Source: PLoS Pathog. 2026 Mar 23;22(3):e1013355. doi: 10.1371/journal.ppat.1013355 (PMC13038106; doi:10.1371/journal.ppat.1013355)

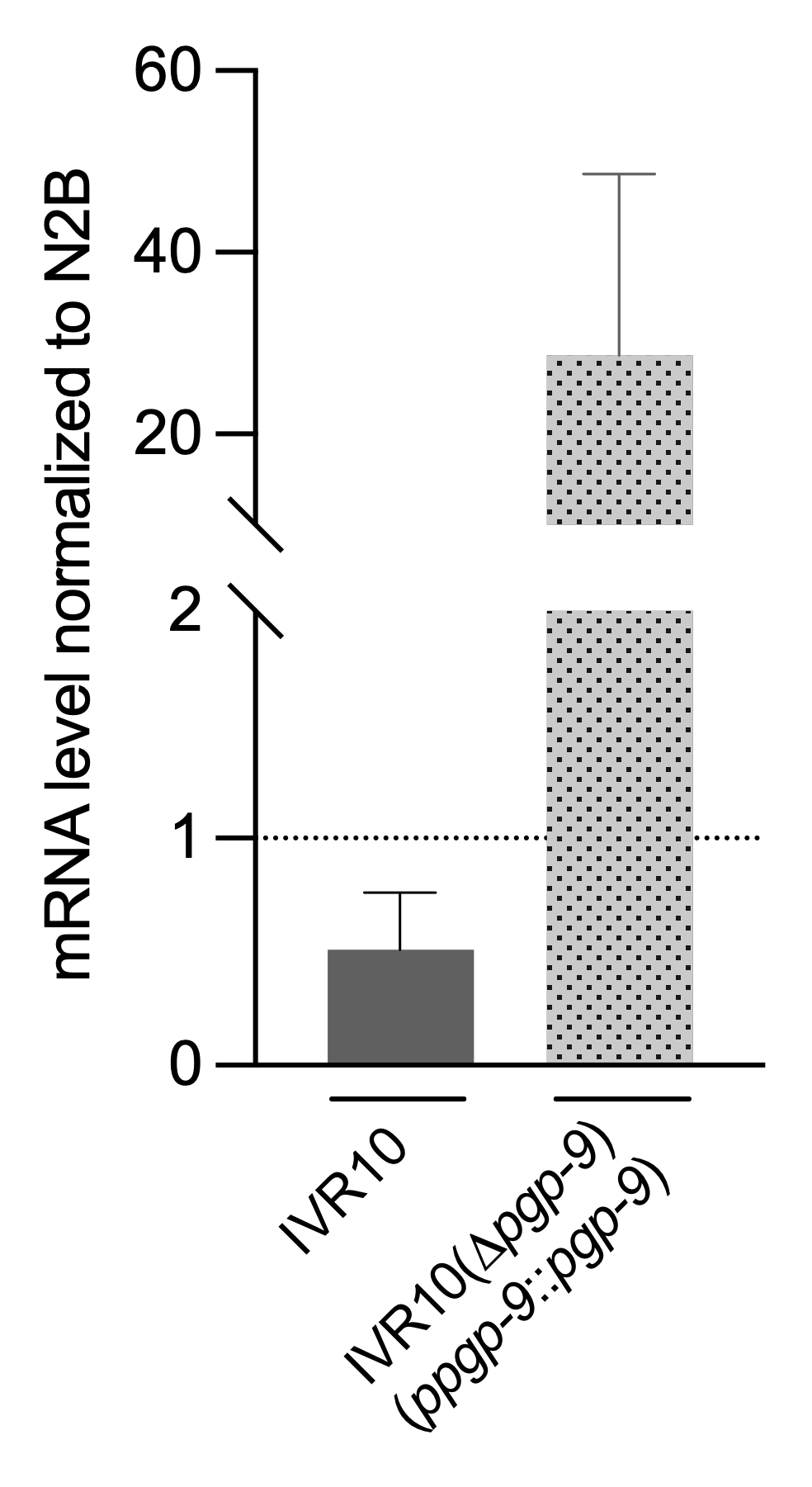

Supplement: S1 Fig — Quantification of Cel-pgp-9 in IVR10(Δpgp-9) and IVR10 by single worm RT-qPCR. Data are expressed as fold change to the expression level of Cel-pgp-9 in the wild-type strain N2B. pgp-9 mRNA levels were normalized against the housekeeping gene tba-1 and are mean ± S.D. from three independent mRNA preparations per strain. One independent mRNA preparation corresponds to an RNA extraction from one single worm. (TIFF) [file ppat.1013355.s001.tiff]

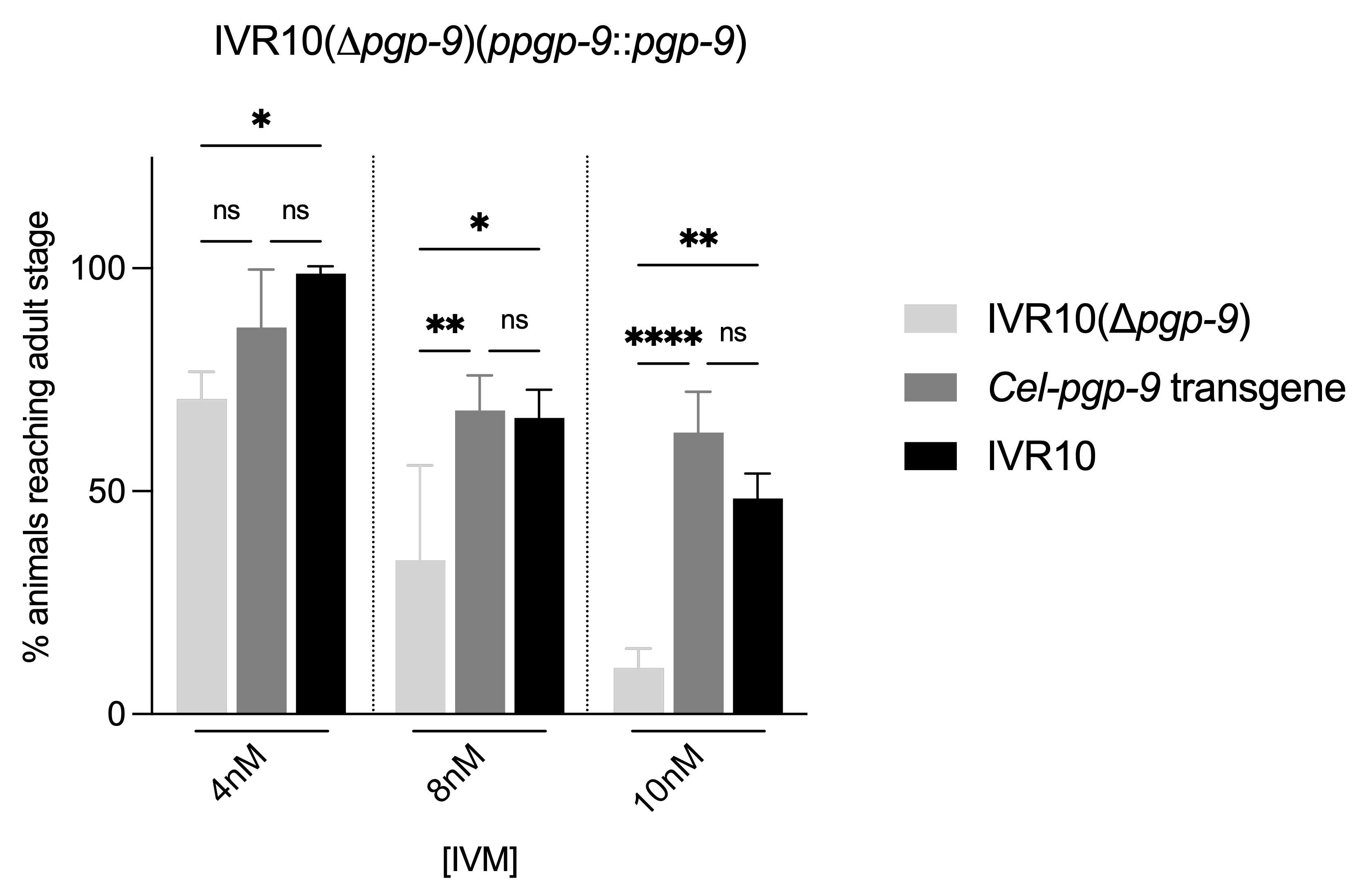

Supplement: S2 Fig — Effect of transgene expression of Cel-pgp-9 on the tolerance of IVR10(Δpgp-9) to ivermectin (IVM) in a modified larval development assay (LDA), with IVR10 as a control. For each IVM concentration, the number of adult worms negative (i.e., IVR10(Δpgp-9)) and positive (i.e., Cel-pgp-9 transgene) for the extrachromosomal array was normalized to the number of adult worms of each population present in the DMSO well and expressed as a percentage. The percentages of animals reaching the adult stage for all concentrations were compared using pairwise using a two-way ANOVA followed by Tukey’s post-hoc multiple comparisons test). Data are mean ± S.D. from 3 independent experiments. Transgenic strain genotype: IVR10[Cel-pgp-9 -; pCel-pgp-9::Cel-pgp-9::3’UTR; pPD118.3]. (TIFF) [file ppat.1013355.s002.tiff]

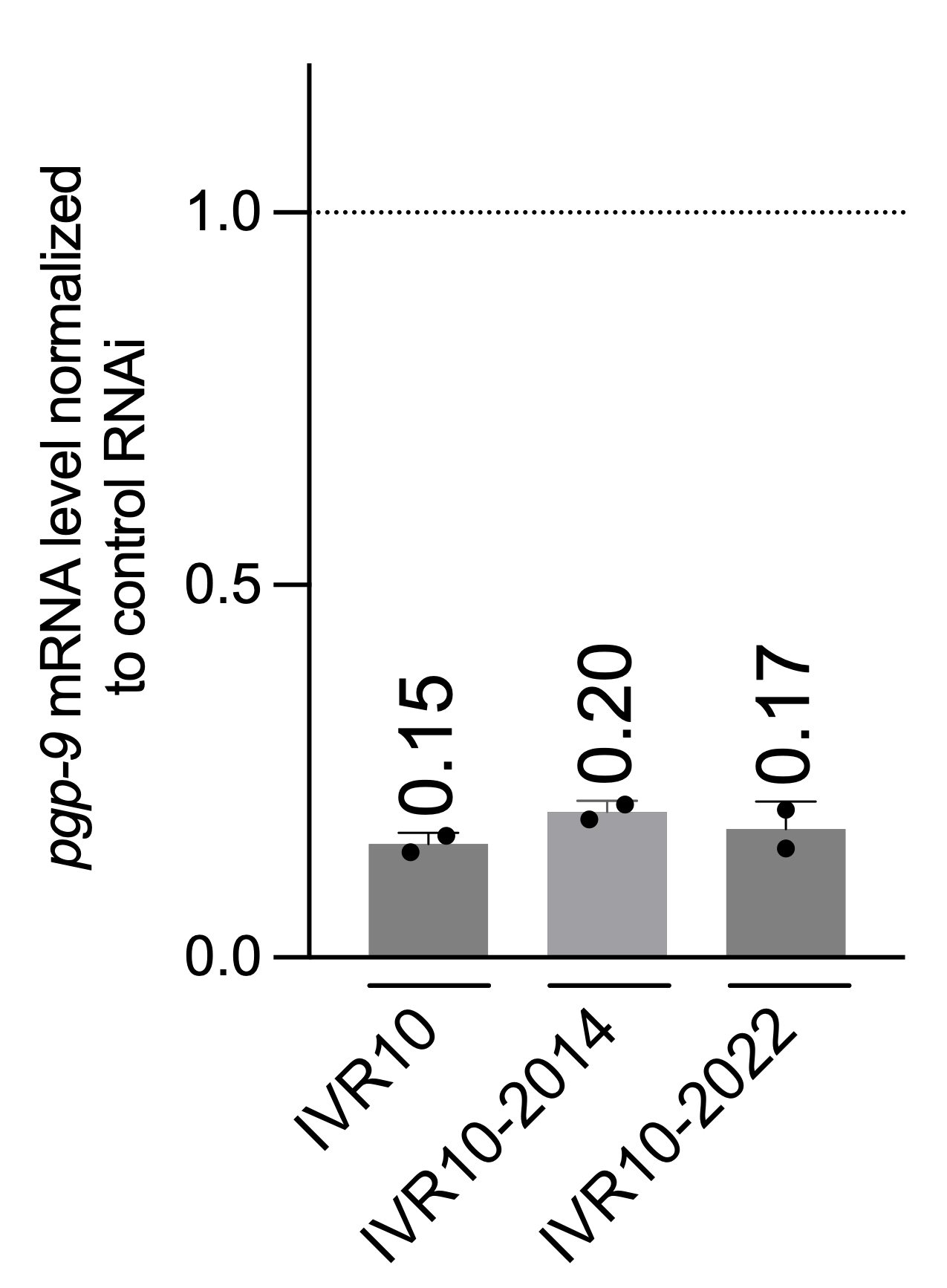

Supplement: S4 Fig — Quantification of pgp-9 transcripts in IVR10, IVR10–2014, and IVR10–2022 following gene silencing. Real-time RT-qPCR analysis was applied after RNAi treatment with control RNAi or specific pgp-9 RNAi. pgp-9 mRNA levels in IVR10 strains are expressed as fold change relative to control RNAi. Data were normalized against tba-1 as an internal control and are mean ± S.D. from two independent RNA preparations for each strain. (TIFF) [file ppat.1013355.s004.tiff]

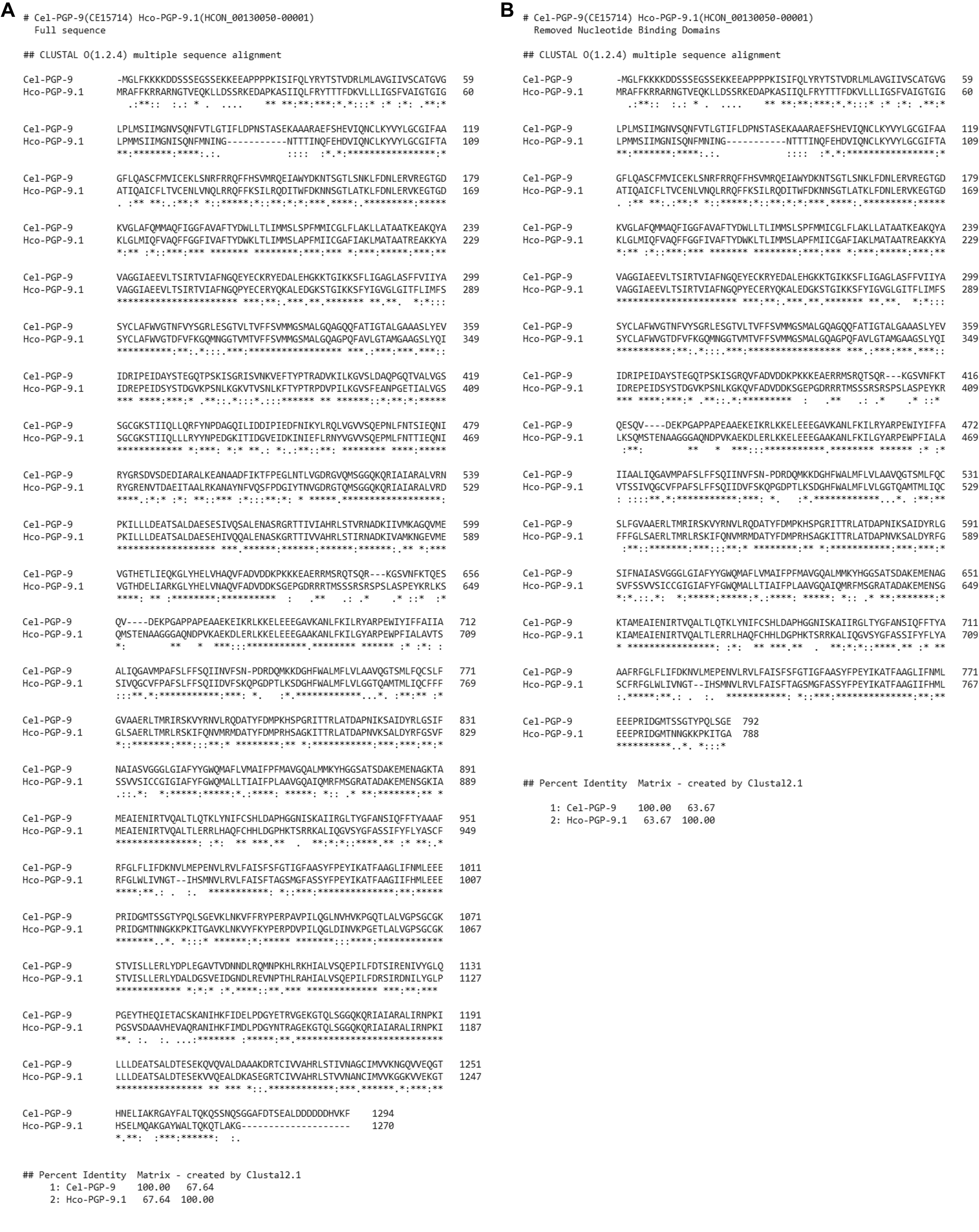

Supplement: S5 Fig — Sequence alignments and percent identity matrices between Cel-PGP-9 (Accession Number: CE15714) and Hco-PGP-9.1 (Accession Number: HCON_00130050–00001) were conducted with the Clustal Omega tool. Symbols indicate: (*) conserved amino acid; (:) strong conservation following a substitution; (.) low similarity following a substitution; () no conservation. (A) Alignment was first performed with full predicted sequences and the percentage of identity between the two proteins was extracted. (B) Nucleotide binding domains (NBDs) were identified with the Scan Prosite tool and then manually curated from the sequences. Sequences were subjected a second time to Clustal Omega in order to highlight homology related to the substrate catalytic part of the protein (i.e., the transmembrane domains (TMDs). (TIFF) [file ppat.1013355.s005.tiff]

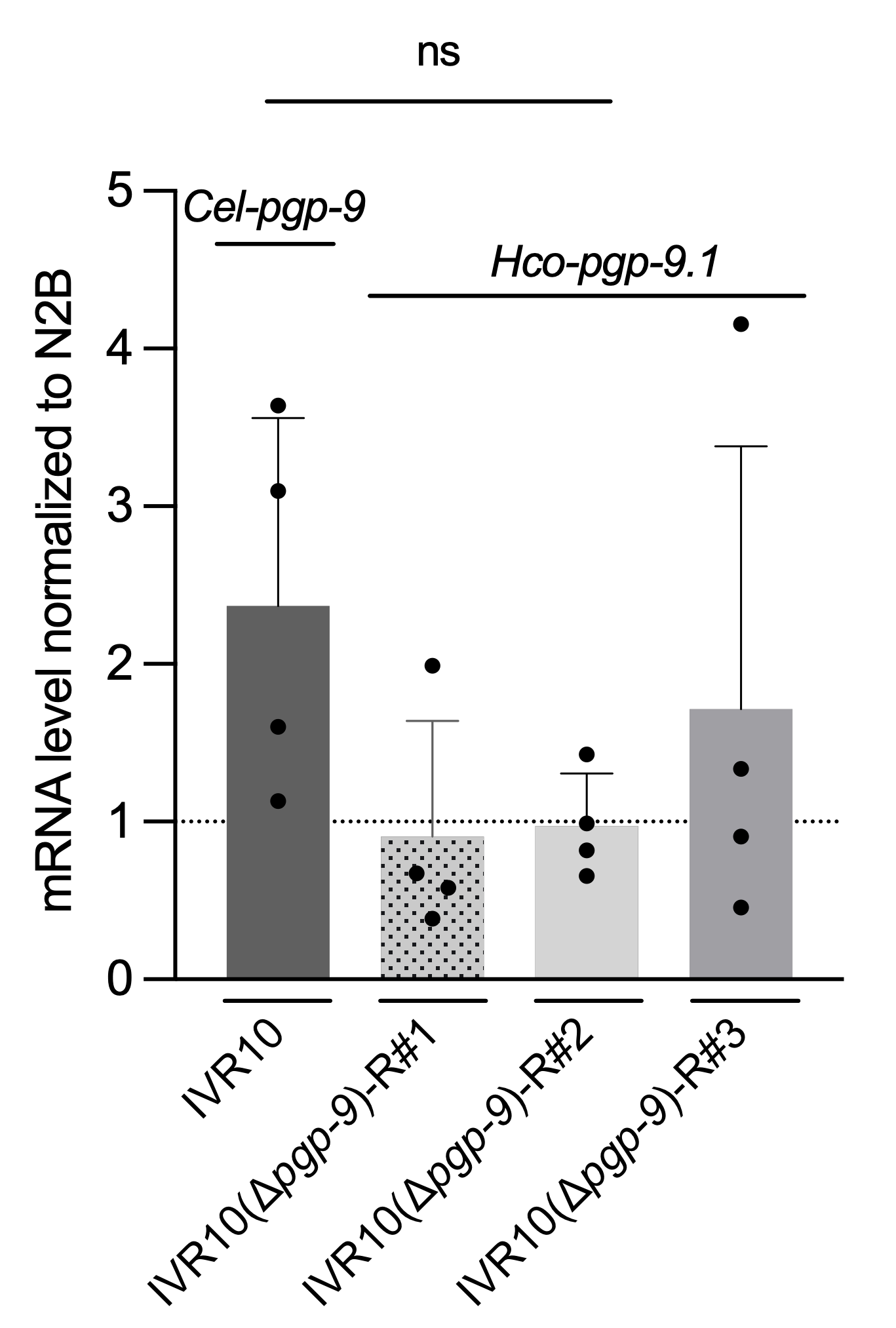

Supplement: S6 Fig — Quantification of Cel-pgp-9 in IVR10 and Hco-pgp-9.1 in the transgenic rescue strains, i.e., IVR10(Δpgp-9)-R#1, #2 and #3, by single worm RT-qPCR. Data are expressed as fold change to the expression level of Cel-pgp-9 in the wild-type strain N2B. pgp-9 mRNA levels were normalized against the housekeeping gene tba-1 and are mean ± S.D. from four independent mRNA preparations per strain. One independent mRNA preparation corresponds to an RNA extraction from one single worm. mRNA levels of Hco-pgp9.1 were compared to those of the IVR10 background strain as a reference (unpaired parametric t-test, ns). (TIFF) [file ppat.1013355.s006.tiff]

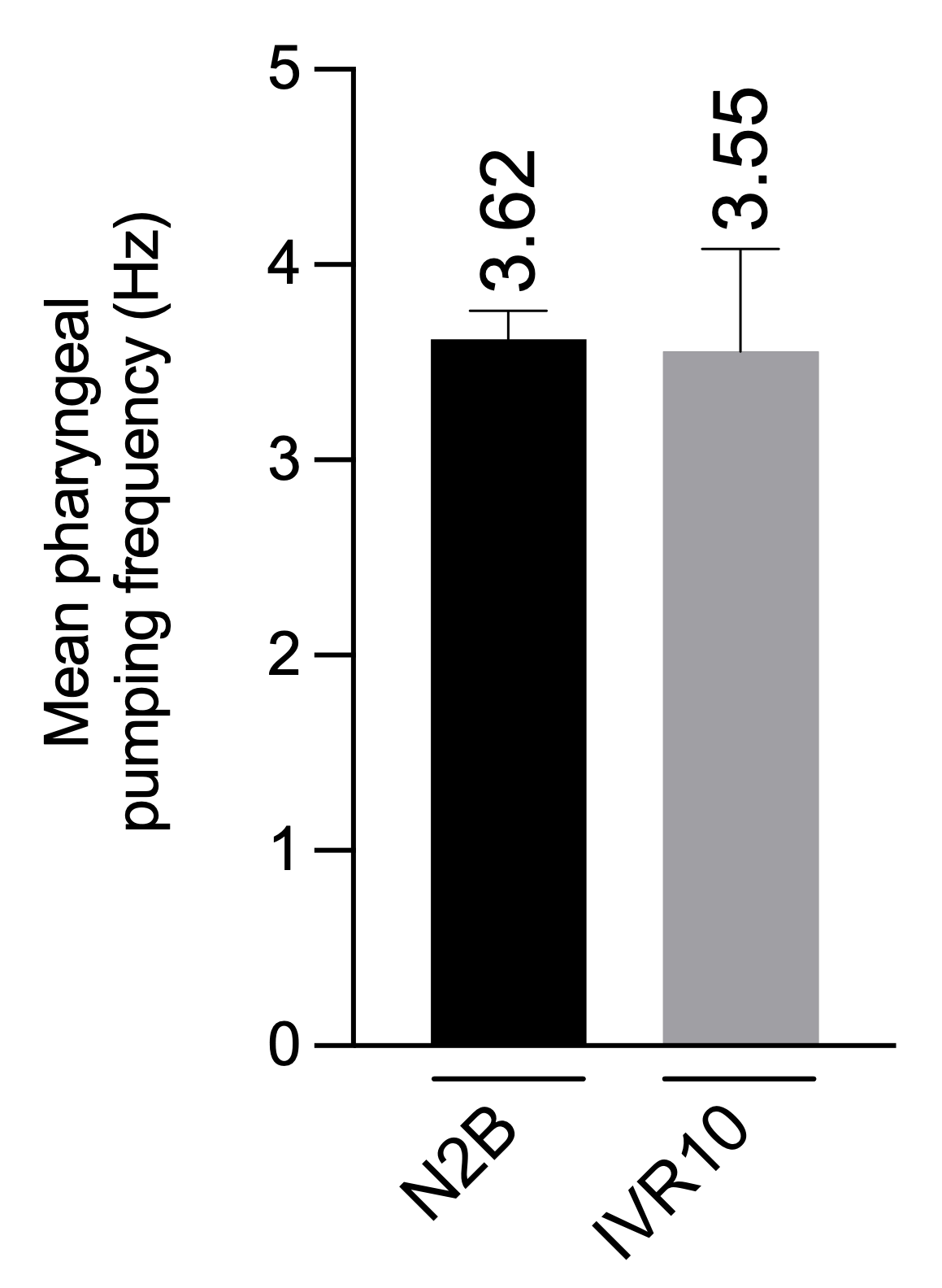

Supplement: S7 Fig — The pharyngeal pumping frequency (Hertz, Hz) corresponds to the average number of pharyngeal pumps recorded per worm for ~1 min. Data are presented as mean ± S.D. from three independent experiments with at least 10 worms per strain per replicate. No significant difference was observed between N2B and IVR10 (unpaired parametric t-test, p > 0.05). (TIFF) [file ppat.1013355.s007.tiff]
