# Supplementary material for: Critical role of P-Glycoprotein-9 in ivermectin tolerance in nematodes
Source: PLoS Pathog. 2026 Mar 23;22(3):e1013355. doi: 10.1371/journal.ppat.1013355 (PMC13038106; doi:10.1371/journal.ppat.1013355)
Supplement: S1 Table — (DOCX) [file ppat.1013355.s009.docx]

**S1 Table. Specific primers for *Caenorhabditis elegans* or *Haemonchus contortus* genes targeted by RT-qPCR.**

| Primer name | Forward | Reverse |
| --- | --- | --- |
| *Cel-pgp-9* | CAAATCTGCTATTGATTATCGGCTC | CCGAGACCTCCACCAACTGA |
| *Cel-tba-1* | ATCGATTTTTGTAGATCTTGAGCCA | TCCAGTGCGGATCTCATCAAC |
| *Hco-pgp-9.1* | GGCCTCAGTTTGCTGTTCTC | ATCTGGTCGCGTTGGATAAG |
