# Supplementary material for: Critical role of P-Glycoprotein-9 in ivermectin tolerance in nematodes
Source: PLoS Pathog. 2026 Mar 23;22(3):e1013355. doi: 10.1371/journal.ppat.1013355 (PMC13038106; doi:10.1371/journal.ppat.1013355)
Supplement: S2 Table — In bold: restriction sites. Underlined: overhangs for Gibson cloning. (DOCX) [file ppat.1013355.s010.docx]

**S2 Table. Primers used for cloning and DNA amplification in this study.**

| Primer name | Forward | Reverse |
| --- | --- | --- |
| pCel9_Blunt^a^ | GGTGATTGGAAGGGAAGTCG | CCGATGATGAATCATCCTTCTTC |
| pCel9^b^ | ATAAGCTTGCATGCCTGCA**G**CCACTGAGCCACTTGGTCAT | GTCCTTTGGCCAATCC**GGG**ACCTGAAAAAAATACATAAAATTC |
| *Cel-pgp-9*^c^ | CGAATTCAACACATTTTTATTACC | CTGACACCACGTGTAGGC |

^a^ primers used for subcloning into pMini 2.0; ^b^ primers used for cloning into pPD95.75. ^c^ primers used for *Cel-pgp-9* amplification, including the promoter and 3’UTR.

In bold: restriction sites. Underlined: overhangs for Gibson cloning.
