# Supplementary material for: Critical role of P-Glycoprotein-9 in ivermectin tolerance in nematodes
Source: PLoS Pathog. 2026 Mar 23;22(3):e1013355. doi: 10.1371/journal.ppat.1013355 (PMC13038106; doi:10.1371/journal.ppat.1013355)
Supplement: S3 Table — IC50: inhibitory concentration 50%. a p < 0.001, b p < 0.05 strain fed on pgp-9 RNAi versus strain fed on control RNAi (unpaired parametric t-test). (DOCX) [file ppat.1013355.s011.docx]

**S3 Table. Susceptibilities to ivermectin (IVM) of IVM selected strains IVR10, IVR10-2014, and IVR10-2022 following *pgp-9* silencing on larval development assay (LDA).** IC_50_: inhibitory concentration 50%.

| Strain | RNAi | Mean IC_50_ (nM)  ± S.D. (no. of experiments) |
| --- | --- | --- |
| IVR10 | control | 11.62 ± 1.75 (3) |
| IVR10 | *pgp-9* | 7.15 ± 0.33 (3)^b^ |
| IVR10-2014 | control | 10.71 ± 0.47 (3) |
| IVR10-2014 | *pgp-9* | 6.81 ± 0.44 (3)^a^ |
| IVR10-2022 | control | 11.37 ± 1.95 (3) |
| IVR10-2022 | *pgp-9* | 6.06 ± 1.01 (3)^b^ |

^a^ p< 0.001, ^b^ p<0.05 strain fed on *pgp-9* RNAi *versus* strain fed on control RNAi (unpaired parametric t-test).
