# Supplementary material for: Critical role of P-Glycoprotein-9 in ivermectin tolerance in nematodes
Source: PLoS Pathog. 2026 Mar 23;22(3):e1013355. doi: 10.1371/journal.ppat.1013355 (PMC13038106; doi:10.1371/journal.ppat.1013355)
Supplement: S4 Table — Calculation method: a ChemPropStd; b CLogP Driver; c Molecular Networks; d Molecular Topology; e MM2 minimization. (DOCX) [file ppat.1013355.s012.docx]

**S4 Table. Comparison of molecular parameters between IVM and F-IVM, as modeled using ChemDraw Professional 17.1 and Chem3D 17.1 (PerkinElmer Informatics).**

| Molecular parameter | IVM | F-IVM |
| --- | --- | --- |
| Chemical formula^a^ | C_48_H_74_O_14_ | C_48_H_70_O_12_ |
| Molecular weight (g/mol)^a^ | 875.1 | 839.1 |
| Number of H bond acceptors^a^ | 13 | 11 |
| Number of H bond donors^a^ | 3 | 1 |
| Log P^b^ | 5.4 | 8.2 |
| Log S^c^ | -5.8 | -8.7 |
| PKa^c^ | 13.7 ; 12.1 ; 15.2 | 13.7 |
| Polar surface area (Å²)^d^ | 170.1 | 129.6 |
| 1.4 Van Der Walls^e^ | 61.0 | 63.1 |

Calculation method: ^a^ ChemPropStd; ^b^ CLogP Driver; ^c^ Molecular Networks; ^d^ Molecular Topology; ^e^ MM2 minimization.
