# Supplementary material for: Critical role of P-Glycoprotein-9 in ivermectin tolerance in nematodes
Source: PLoS Pathog. 2026 Mar 23;22(3):e1013355. doi: 10.1371/journal.ppat.1013355 (PMC13038106; doi:10.1371/journal.ppat.1013355)
Supplement: S5 Table — (DOCX) [file ppat.1013355.s013.docx]

**S5 Table. Evidence of *Cel-pgp-9*/PGP-9 localization from the literature.**

| Localization | Method | Reference |
| --- | --- | --- |
| Pharynx bulbs, gut | GFP promoter fusion | [38] |
| Neurons: I1, NSM, I6, PQR, RIH, URX, PLM, AIA | Single-cell RNA-sequencing (scRNA-seq) of mature *C. elegans* nervous system | CeNGEN[40] |
| Intestine (intestinal rectal valve, intestine anterior, rectal gland), coelomocytes, pharynx (pharyngeal muscle, marginal cells), neurons (URX, AQR, PQR), arcade cells | scRNA-seq of young adults | WORMSEQ[37] |
| Intestine (anterior, posterior, middle), coelomocytes, pharynx, PVD neurons (AQR, PQR, URX) | scRNA-seq of L2 | VisCello - *C. elegans* L2 Data[41] |
| Alimentary (intestine) and nervous (head, ciliated, sensory, tail, and inter-neurons) systems, pharynx, coelomocytes | Pooled analysis of tissue-specific transcriptome data, *i.e.*, 4,342 microarrays and RNA-seq across 273 datasets of adult stage *C. elegans* | [42] |
