# Supplementary material for: Critical role of P-Glycoprotein-9 in ivermectin tolerance in nematodes
Source: PLoS Pathog. 2026 Mar 23;22(3):e1013355. doi: 10.1371/journal.ppat.1013355 (PMC13038106; doi:10.1371/journal.ppat.1013355)
Supplement: S1 File — Multiple sequence alignment was performed with the Clustal Omega method using Seaview and served as the basis for the phylogenetic tree. Cel-pgp-15 is a pseudogene and was therefore not included. (PDF) [file ppat.1013355.s014.pdf]

```
                                    1
Cel-PGP-1,CE11932                   ---------- ---------- -------MLR NGSLR-Q--- -----SLRTL
Cel-PGP-2,CE41207                   ---------- ---------- ---------- ---------- ----------
Cel-PGP-3,CE03818                   ---------- ---------- ---------- ---------- ----------
Cel-PGP-4,CE44238                   ---------- ---------- ---------- ---------- ----------
Cel-PGP-5,CE43003                   ---------- ---------- ---------- ---------- ----------
Cel-PGP-6,CE40818                   ---------- ---------- ---------- ---------- ----------
Cel-PGP-7,CE53605                   ---------- ---------- ---------- ---------- ----------
Cel-PGP-8,CE31624                   ---------- ---------- ---------- ---------- ----------
Cel-PGP-9,CE15714                   ---------- ---------- ---------- ---------- ----------
Cel-PGP-10,CE40807                  ---------- ---------- -MSNEKNKNE STNKTNEIGN AE--------
Cel-PGP-11,CE53819                  ---------- ---------- ---------- ---------- ----------
Cel-PGP-12,CE03260                  ---------- ---------- ------MKWR ------KNGD ----------
Cel-PGP-13,CE53174                  ---------- ---------- -MPPQENT-D ------GSGK NL---S----
Cel-PGP-14,CE03262                  ---------- ---------- -MAPKDDP-D ------NRGF DD---Q----
Hco-PGP-1,HCON_00098130-00001       ---------- ---------- ---------- ---------- ----------
Hco-PGP-2,HCON_00004450-00001       ---------- ---------- ---------- ---------- ---------R
Hco-PGP-3,HCON_00042800-00001       ---------- ---------- ---------- ---------- ----------
Hco-PGP-9.1,HCON_00130050-00001     ---------- ---------- ---------- ---------- ----------
Hco-PGP-9.2,HCON_00130060-00001     ---------- ---------- ---------- ---------- ----------
Hco-PGP-10,HCON_00168800-00001      ---------- ---------- -MT------- -------AKK DA--------
Hco-PGP-11,HCON_00162780-00001      ---------- ---------- ---------- ---------- ----------
Hco-PGP-13,HCON_00041390-00001      ---------- ---------- -MTPKPDFYL ------QRGT ADPQRS----
Hco-PGP-16,HCON_00035895-00001      ---------- ---------- ---------- ---------- ----------
Hsa-ABCB1,NP_001335874.1            MSVNLQGDQR GATEARTFLL EIQPVSQFLE ESAFS-QSGP GAVICGLSTK
Mmu-ABCB1,NP_035205.1               ---------- ---------- ---------- ---------- ----------

                                   51
Cel-PGP-1,CE11932                   DS------FS LAPE-DV--- LKTAIKTVED Y--------- -------EGD
Cel-PGP-2,CE41207                   ---------- MGK--DT--- EKTPLLLKSL ---------- -------DKQ
Cel-PGP-3,CE03818                   ---------- ---------- ----MKKTKV N--------- --------PE
Cel-PGP-4,CE44238                   ---------- MPEATDA--- LLEPVVPMKI A--------- ---------S
Cel-PGP-5,CE43003                   ---------- ------M--- DSFPLLPFS- ---------- ----------
Cel-PGP-6,CE40818                   ---------- MEENSNN--- FSTPLLPQS- ---------- ----------
Cel-PGP-7,CE53605                   ---------- MEESSND--- YTTPLIAQS- ---------- ----------
Cel-PGP-8,CE31624                   ---------- ME---NS--- ENDVLIPVE- ---------- ----------
Cel-PGP-9,CE15714                   ---------- ---------- -----MGLFK K--------- -------KKD
Cel-PGP-10,CE40807                  -----KKEEA ETEDKKSLKS MIYPIEPSTL ---------- ------YIDD
Cel-PGP-11,CE53819                  ---------- ---------- ---------- ---------- ----------
Cel-PGP-12,CE03260                  ---------- -----AI--- EPIDGIPMEN GKKKDTTVNV STEDVGSIDS
Cel-PGP-13,CE53174                  --------SL ---RT----- ----KIDFD- ----EKISAT SFSDSSYNDD
Cel-PGP-14,CE03262                  --------RR PSQRSTV--- LAIPALAVND PK--SDPKIA SDLPANYVDD
Hco-PGP-1,HCON_00098130-00001       ---------- ---------- ---------- ---------- ---------M
Hco-PGP-2,HCON_00004450-00001       DRSQAGKMFE KGQ--DD--- ERIPLLGSSK ---------- -------K--
Hco-PGP-3,HCON_00042800-00001       ---------- ---------- -------MKI S--------- --------PL
Hco-PGP-9.1,HCON_00130050-00001     ---------- ---------- ----MRAFFK R--------- -------RAR
Hco-PGP-9.2,HCON_00130060-00001     ---------- ---------- -----MGFLK K--------- -------KGK
Hco-PGP-10,HCON_00168800-00001      -----KKKDP PNEESPARKN VVYPIQ--DL ---------- ------YVND
Hco-PGP-11,HCON_00162780-00001      ---------- ---------- ---------- ---------- -------MTA
Hco-PGP-13,HCON_00041390-00001      --------SE TSKKSTV--- LAVPA----- ---------- --FASPVEDD
Hco-PGP-16,HCON_00035895-00001      ---------- MGD------- ------DAKK ---------- ---------D
Hsa-ABCB1,NP_001335874.1            VGVSSSKISR LGGR-SK--- EREVGMDLEG DRNG------ -----GAKKK
Mmu-ABCB1,NP_035205.1               ---------- ---------- -----MEFEE NL-K------ -----GRADK
```

```
                                  101
Cel-PGP-1,CE11932                 NIDSNGE--- ---------- -IKITRDAK- -EEVVNKVSI PQLYRYTTTL
Cel-PGP-2,CE41207                 RSQTSLS--- ---------- -SHSSDSSI- -DESTVKLTN YGIFYYTQGV
Cel-PGP-3,CE03818                 DDITLGK--- ---------- -FTP--KPS- -PQDSYQGNF FDVFRDADYK
Cel-PGP-4,CE44238                 EHVELGS--- ---------- -RPDKKKKK- -SRSSQGNSL SNLFRHSGCA
Cel-PGP-5,CE43003                 ---------- ---------- ------SNT- -NWSNVCKFI KVIIKCTTRY
Cel-PGP-6,CE40818                 ---------- ---------- ------DEK- -KPSQICSFI RVVFKCSSCF
Cel-PGP-7,CE53605                 ---------- ---------- ------NEK- -KPLELCSFI RVIFKCTSCF
Cel-PGP-8,CE31624                 ---------- ---------- ------NEK- -KTTNWTKFV KVVWQCTSKW
Cel-PGP-9,CE15714                 DSSSE----- ---------- -GSSEKKEE- -APPPPKISI FQLYRYTSTV
Cel-PGP-10,CE40807                LKNSKKAYDV TMDDPL---D ---KSSDSK- -PKLGFFEPY KRLYAFADSI
Cel-PGP-11,CE53819                ---------M KWIKKLCRPV SAFGDESKVS DVTPSIRQSL NLMINFAKKS
Cel-PGP-12,CE03260                DEEPKIVYEP SKMEKLINYM LCRGDLASR- -ELSVKPVTL LGLFRYAERT
Cel-PGP-13,CE53174                DNDKKVEYYP SVFEKFVNFL LCRCDLSEQ- -VLEFQPVSL LQLFRFATTF
Cel-PGP-14,CE03262                DDDAPKMYTP SLLEKILNYA LCRGDIANQ- -QLEAQPVSI PGLFRYGKKF
Hco-PGP-1,HCON_00098130-00001     GFRKNTK--- ---------- -KDEKAVVK- -EEPAKKVPL LQLWRYATWS
Hco-PGP-2,HCON_00004450-00001     ---------- ---------- -SSIGEVSK- -KEEPPTITN RGILSLATTL
Hco-PGP-3,HCON_00042800-00001     EKVRN----- ---------- ------TKR- -KKAPRQAGL LDIMRGASCL
Hco-PGP-9.1,HCON_00130050-00001   NGT--VE--- ---------- -QKLLDSSR- -KEDAPKASI IQLFRYTTTF
Hco-PGP-9.2,HCON_00130060-00001   AADSKES--- ---------- -ENDNEEEK- -KDDAPKASI PQLFRYTTTF
Hco-PGP-10,HCON_00168800-00001    PKKNAKTYKV NSENKV---M VENESKETK- -KQKDFFSSL AVLYGFSDRT
Hco-PGP-11,HCON_00162780-00001    IKEEEGSISR TFFEKLLDVL LCRGDLSTE- -KLERKPVSV AELFRYASRR
Hco-PGP-13,HCON_00041390-00001    DDDRKYTYTP STIEKVINIL LCRGDLANR- -VLEVKPVSI FGLFRYATKW
Hco-PGP-16,HCON_00035895-00001    QQEKLS---- ---------- -----EKTP- -AEKPARVPL PVLFRYATKL
Hsa-ABCB1,NP_001335874.1          NFF--KL--- ---------- -NNKSEKDK- -KEKKPTVSV FSMFRYSNWL
Mmu-ABCB1,NP_035205.1             NFS--KM--- ---------- -GKKSKKEK- -KEKKPAVGV FGMFRYADWL

                                  151
Cel-PGP-1,CE11932                 EKLLLFIGTL VAVITGAGLP LMSILQGKVS QAFINEQIVI N-NNGSTF--
Cel-PGP-2,CE41207                 DLLLLITGTV AAVIHGAGFP LLAIVLGGMT TVFLRAQNSD F-VVGVD--N
Cel-PGP-3,CE03818                 DYILFSGGLI LSAVNGALVP FNSLIFEGIA NALMEGESQY ----------
Cel-PGP-4,CE44238                 DYLLLLGGLV LSAANGALLP FNSLIFEGIT NVLMKGEAQW ----------
Cel-PGP-5,CE43003                 EKLLFFLGVV FSILTGMCQP FESYTLGETS QVLVKVTNAI N-NK------
Cel-PGP-6,CE40818                 EKFLFLLGVL FSLMTGFCQP FMSYTFGEVS QVLVTITNAI N-NK------
Cel-PGP-7,CE53605                 EKFLFLIGVF FSLVTGLCQP FVSYTLGETA QVLVTITNAI N-NK------
Cel-PGP-8,CE31624                 EKFLFVIGVV SAICTGLTQP FMSYTFGEVS QAFVRITAAV N-NA------
Cel-PGP-9,CE15714                 DRLMLAVGII VSCATGVGLP LMSIIMGNVS QNFVTLGTIF L-DPNST---
Cel-PGP-10,CE40807                DMQLMAMGLF SALLQSALPP FVWLIMGNFV SISILREEGK L-GLKN----
Cel-PGP-11,CE53819                DLIFIITALI CALLGGTIQP VVLLIGGWIT DLYLTNGNTA ----------
Cel-PGP-12,CE03260                DYALLCFGIF LSFISGIAQP GLGIIAGNIT NSLLIHNATS ----------
Cel-PGP-13,CE53174                DYILLLIGLI TSVISGVSQP VLAIISGRMT NVLLVIDPLS ----------
Cel-PGP-14,CE03262                DYLLLFIGTI CAIISGVSQP ILALVSGRVT NALLVYPPTS ----------
Hco-PGP-1,HCON_00098130-00001     ELALLFVGIF VSLVTGAGLP LMSIIQGQVT QAFVKEEMYK -----THT--
Hco-PGP-2,HCON_00004450-00001     DYVLLAVGTL ASCVHGAGFS VLGIVLGGMT TVFLRAQNSE F-VLGTV--S
Hco-PGP-3,HCON_00042800-00001     DYVFLLLGVV FSLVNGAILP LNSLVFQGMA DTLITGERNH ----------
Hco-PGP-9.1,HCON_00130050-00001   DKVLLLIGSF VAIGTGIGLP MMSIIMGNIS QNFMNING-- ----------
Hco-PGP-9.2,HCON_00130060-00001   DKLLLLIGST VAMGTGMGLP MMAIIMGDIT QNFMSVNG-- ----------
Hco-PGP-10,HCON_00168800-00001    DIQLMSIGLT CALMQSVIPP FVWLVMGSFV SFSITREEER LYNISG----
Hco-PGP-11,HCON_00162780-00001    DCHYVAAGFV LAVVVGAIMP LTCIFGGLYV NIYLMNTDHV ----------
Hco-PGP-13,HCON_00041390-00001    DRFCIFIGVI CSIISGVSQP IMALVSGRVT NVLLVYPPNS ----------
Hco-PGP-16,HCON_00035895-00001    DFCLMLLGAV FAATQGTFNS VSSLVFRHLM DALIIGEFEW ----------
Hsa-ABCB1,NP_001335874.1          DKLYMVVGTL AAIIHGAGLP LMMLVFGEMT DIFANAGNLE D-LMSNITNR
Mmu-ABCB1,NP_035205.1             DKLCMILGTL AAIIHGTLLP LLMLVFGNMT DSFTKAEA-- S-ILPSITNQ
```

```
                                  201
Cel-PGP-1,CE11932                 --LPTGQNYT KTDFEHDVMN VVWSYAAMTV GMWAAGQITV TCYLYVAEQM
Cel-PGP-2,CE41207                 VNPEGLVPIS LDEFNSEVVK YCIYYLVLGV LMFFTSYVQI ACFESYAERL
Cel-PGP-3,CE03818                 ----QNGTIN MPWFSSEIKM FCLRYFYLGV ALFLCSYFAN SCLYTLCERR
Cel-PGP-4,CE44238                 ----QNGTFD YDTFSSGIQH YCLLYFLLGV LMFTCTYFSN ACLFTMAERR
Cel-PGP-5,CE43003                 TIDPVDLAHA YKLFESDMNR VVLLFFLVGF AYFTFGFLQF SIMKFVGDNT
Cel-PGP-6,CE40818                 TIDPADLEKA YEEYERGMNQ VVFHFFLCGC AYFIFASLQH AIMKYVGDNT
Cel-PGP-7,CE53605                 TIDPADLKKA YEQYERGMYQ VVLYFFLCGC AYFTFASIQH AIMKYVGDNT
Cel-PGP-8,CE31624                 SLDPSDLEKA YEMFHADMNN VVIHFGLVGC AFMFFGFIQF SLFKYIGDNT
Cel-PGP-9,CE15714                 ---ASEKAAA RAEFSHEVIQ NCLKYVYLGC GIFAAGFLQA SCFMVICEKL
Cel-PGP-10,CE40807                ---LTSDYPI DDQFAHSATP AFISMLGLSI AMFIAAFCQR IAWEISSIRQ
Cel-PGP-11,CE53819                ---------G NDEFLYSVLT LIYAGLGFGV IILVLALIQG VCIQRGTSRI
Cel-PGP-12,CE03260                ---------- -DDFYDSAMT NVWLFGGIGI IVLIVNFVQY MCFQYCCIRI
Cel-PGP-13,CE53174                ---------- -KEFKTKTME NVYIFLGLGI FVSINDFCQY MCFQRVCSRM
Cel-PGP-14,CE03262                ---------- -KQFRNKANE NVYIFLGIGI FISITNFIQY MCFQHCCTRV
Hco-PGP-1,HCON_00098130-00001     --LPGPIHYN DTDFTNDVMN AVYGYTGMAV GMFIAANVQV TCFLIVCEQM
Hco-PGP-2,HCON_00004450-00001     RDPEGLPALT KEEFDTLVRR YCLYYLGLGF AMFATSYIQI VCWETFAERI
Hco-PGP-3,HCON_00042800-00001     ----SRNELD MDVFTANVLL YCGLYLGLGI ALLAIGYISN ASLYTLCERR
Hco-PGP-9.1,HCON_00130050-00001   ------NTTT INQFEHDVIQ NCLKYVYLGC GIFTAATIQA ICFLTVCENL
Hco-PGP-9.2,HCON_00130060-00001   ------NRTS FQQFEHDVTQ NCLKYVYLGC GIFAAATIQA TCFLTVGENL
Hco-PGP-10,HCON_00168800-00001    ---AANGSKF DADFAASATP AFIIMLSLSV SMFSAAFIQR LAWEVSGIRQ
Hco-PGP-11,HCON_00162780-00001    ---------G NESLWRQAMY LCAGYFGVGI ALFILCYLQN YFLSLASHNI
Hco-PGP-13,HCON_00041390-00001    ---------- -KEFRNKAYE NVYIFLGIGV FVLITNFIQF MCFHSCCTRV
Hco-PGP-16,HCON_00035895-00001    ----QAGIFD DYEFTQLAMN SVYRYTLFGL IQFTLGFLSM CCWHTVCERQ
Hsa-ABCB1,NP_001335874.1          SDINDTGFF- -MNLEEDMTR YAYYYSGIGA GVLVAAYIQV SFWCLAAGRQ
Mmu-ABCB1,NP_035205.1             SGPNSTLIIS NSSLEEEMAI YAYYYTGIGA GVLIVAYIQV SLWCLAAGRQ

                                  251
Cel-PGP-1,CE11932                 NNRLRREFVK SILRQEISWF DTNHSGTLAT KLFDNLERVK EGTGDKIGMA
Cel-PGP-2,CE41207                 VHKLRQNYLK AILRQQIQWF DKQQTGNLTA RLTDDLERVR EGLGDKFALL
Cel-PGP-3,CE03818                 LHCIRKKYLK SVLRQDAKWF DETTIGGLTQ KMSSGIEKIK DGIGDKVGVL
Cel-PGP-4,CE44238                 LYCIRKHLLQ SVLRQDAKWF DENTVGGLTQ KMSSGIEKIK DGIGDKIGVL
Cel-PGP-5,CE43003                 AYNVRRQYIS RLLRKDISYF DGMSTGHLSI VLNDNMERFR EVFNEKIALI
Cel-PGP-6,CE40818                 TYRVRKQYIS RLLKKDAQYF DSVSTGHLST VLNDNLERFR EVFNEKIALI
Cel-PGP-7,CE53605                 TYRVRKQYIS RLLRKDAEYF DNVSTGHLST VLNDNLERFR EVFNEKIALI
Cel-PGP-8,CE31624                 TYRLRHKFIL RLLKKDAKYF DTISTGYLST VLNDNLERFR EAFNEKIAFI
Cel-PGP-9,CE15714                 SNRFRRQFFH SVMRQEIAWY DKNTSGTLSN KLFDNLERVR EGTGDKVGLA
Cel-PGP-10,CE40807                VFRIKKAYIR KLLLMDISWL ESRQSGHVAA MLQESADSIY NGISDHLPMV
Cel-PGP-11,CE53819                LDSIRKEFLG AVLRQDANWL DKHSSGSITC QLNENIEVIS DGLGNKCCML
Cel-PGP-12,CE03260                TSKMKQHYIQ SILRQNAGWF DKNHSGTLTT KLHDNMERIN EGIGDKLGVL
Cel-PGP-13,CE53174                MTVMRNRYIS SILRQNAGWF DKNLSGTITT RLNDNMERIQ DGVGDKLGVL
Cel-PGP-14,CE03262                MAQMRHRFVY SVLRQNAGWF DKNHSGTITT KLNDSMERIR EGIGDKLGVL
Hco-PGP-1,HCON_00098130-00001     SNRIRRKFVK AILHQDISWF DKNNSGTLAT KLFDNIERVK EGTGDKIGLI
Hco-PGP-2,HCON_00004450-00001     THKLRKIYLK AILRQQISWF DIQQTGNLTA RLTDDLERVR EGLGDKLSLF
Hco-PGP-3,HCON_00042800-00001     IHSIRARYLR AVLRQDMTWL DQQQTGALTM KMSSGMERIK DGIGDKLGLI
Hco-PGP-9.1,HCON_00130050-00001   VNQLRRQFFK SILRQDITWF DKNNSGTLAT KLFDNLERVK EGTGDKLGLM
Hco-PGP-9.2,HCON_00130060-00001   VNQLRRQFFK SILRQDIPWF DKNGSGTLAT KLFDNLERVK EGTGDKVGLM
Hco-PGP-10,HCON_00168800-00001    VFRVRRTYVR KMLHMDVSWL ESRQSGQMAT MLQEYTDTIY NGISDNIPMV
Hco-PGP-11,HCON_00162780-00001    VGRIRKEFVK AVLAQNAAWF DENNAGTITT KLNENVAQIE DGIGDKIGML
Hco-PGP-13,HCON_00041390-00001    ISKMRHEYVR AILRQNAGWF DRNHSGALST KLNDNMERIR EGIGDKLGLL
Hco-PGP-16,HCON_00035895-00001    VYQIRNRFFG SVIRQDMAWF DQNDSGALTT RMSDGIDRIR DGIGDKLGAM
Hsa-ABCB1,NP_001335874.1          IHKIRKQFFH AIMRQEIGWF DVHDVGELNT RLTDDVSKIN EGIGDKIGMF
Mmu-ABCB1,NP_035205.1             IHKIRQKFFH AIMNQEIGWF DVHDVGELNT RLTDDVSKIN DGIGDKIGMF
```

```
                                    301
Cel-PGP-1,CE11932                   FQYLSQFITG FIVAFTHSWQ LTLVMLAVTP IQALCGFAIA KSMSTFAIRE
Cel-PGP-2,CE41207                   VQMFAAFLAG YGVGFFYSWS MTLVMMGFAP LIVLSGAKMS KSMATRTRVE
Cel-PGP-3,CE03818                   VGGVATFISG VSIGFYMCWQ LTLVMMITVP LQLGSMYLSA KHLNRATKNE
Cel-PGP-4,CE44238                   VSGIATFISG VALGFYMCWQ LTLVMLVTVP LQLGSMYLSA KHLNRATKNE
Cel-PGP-5,CE43003                   IALLTDFVIG TILAFYTDWR LACYGTVFSF GIVLSGLLDS WGKMKNNEKQ
Cel-PGP-6,CE40818                   IAFVTDFTLG TALAFYTDWR LASYGIFFSL GIAFSGFINS AGVMKTTGKQ
Cel-PGP-7,CE53605                   FALLTDFVVG TALAFYTDWR LASYGIFFSL GIAFSGFINS AGVMKTTGKQ
Cel-PGP-8,CE31624                   ICFSTDFAIG TALAFYTSWT LASYGSVFAF GIVISGLLNS TSMMKSNEKQ
Cel-PGP-9,CE15714                   FQMMAQFIGG FAVAFTYDWL LTLIMMSLSP FMMICGLFLA KLLATAATKE
Cel-PGP-10,CE40807                  VFILSYLLVN IGVCLYIQWD VTLFMCLAIP LLIISRILYS KWFANTMEEE
Cel-PGP-11,CE53819                  VRGFAMFTSS IIACAFINWQ LTFITFTMGP VSAFVLHLLT KVNEVSNEEL
Cel-PGP-12,CE03260                  IRGMVMFVAG IVISFFYEWR LALMMMGIGP LCCVCMSLMS RSMSSFTSKE
Cel-PGP-13,CE53174                  IRGISMVIAS VVISLIYEWR LALMMLGLIP VSTICMTLLS RFLEKSTGEE
Cel-PGP-14,CE03262                  LRGFAMLIAA IVVAYIYEWR LASMMLGVAP TCCICMSLLA RQMTSTTIKE
Hco-PGP-1,HCON_00098130-00001       FQYTSQFVTG FVIAFTHSWK LTLVMLAVTP LQAFCGFAIS KSMSTFTIAE
Hco-PGP-2,HCON_00004450-00001       IQMMSAFVAG FCVGFAYSWS MTLVMMVVAP FIVISANWMS KIVATRTQVE
Hco-PGP-3,HCON_00042800-00001       LASIGSFIGG ISLGFYLSWR MTLVMLITVP LLIGATQFSG KLLSRASKME
Hco-PGP-9.1,HCON_00130050-00001     IQFVAQFFGG FIVAFTYDWK LTLIMMSLAP FMIICGAFIA KLMATAATRE
Hco-PGP-9.2,HCON_00130060-00001     IQYVAQFFGG FIVAFTYDWK LTLIMMSLAP FMIMCGAFIA RLMATAATRE
Hco-PGP-10,HCON_00168800-00001      IFISAYLVAN IAVCLYIQWD VTLLMCSAIP MLILSRIVFS KWFSKTMDQE
Hco-PGP-11,HCON_00162780-00001      ARGVTVFIAS AAFAFYYDWR ITLVCIWDGP VSAITMAIMS RLSSPPMQGM
Hco-PGP-13,HCON_00041390-00001      LRGCAMFTAA VIIAFIYEWR LALMMLGVTP TTCAIMSIMA RKMTSTTMRE
Hco-PGP-16,HCON_00035895-00001      FAYFATFIAG ITVALSCSWQ MTLVMIGFFP IFFGPLTVTS MIMGKVVPKE
Hsa-ABCB1,NP_001335874.1            FQSMATFFTG FIVGFTRGWK LTLVILAISP VLGLSAAVWA KILSSFTDKE
Mmu-ABCB1,NP_035205.1               FQSITTFLAG FIIGFISGWK LTLVILAVSP LIGLSSALWA KVLTSFTNKE

                                    351
Cel-PGP-1,CE11932                   TLRYAKAGKV VEETISSIRT VVSLNGLRYE LERYSTAVEE AKKAGVLKGL
Cel-PGP-2,CE41207                   QETYAVAGAI AEETFSSIRT VHSLNGHKRE LDRFYNALEV GRQTGIVKYC
Cel-PGP-3,CE03818                   MSAYSNAGGM ANEVIAGIRT VMAFNAQPFE INRYAHQLNE ARRMGIRKAI
Cel-PGP-4,CE44238                   MSAYSSAGGM ANEVIAGIRT VIAFNAQPFE IERYGAQLAK ARKMGIRKAI
Cel-PGP-5,CE43003                   NEHISNAGSI AFQALGCYKT VSSLNGQQQE VERYTEELKN GEKYALNRAF
Cel-PGP-6,CE40818                   NTHYANAGSI AFQTLGAYKT VCSLNGQKTE IERYTEELKA GEKYGIRRAL
Cel-PGP-7,CE53605                   NTHYANAGSI AFQTLGAYKT VCSLNGQNTE IERYTEELKA GEKYGIHRAL
Cel-PGP-8,CE31624                   SMHYSNAGAI AFQALCSFKT VISLNGQTQE LEKYSAELKE GEKFGSRRAF
Cel-PGP-9,CE15714                   AKQYAVAGGI AEEVLTSIRT VIAFNGQEYE CKRYEDALEH GKKTGIKKSF
Cel-PGP-10,CE40807                  TKLQNKITNL VNETFNCITT VISFAAQKQK INKFEKLSAE HSKLTEERLR
Cel-PGP-11,CE53819                  MSLSAQSHAI IEESILNVRT VQSCNGQNFM ITKLNQVNEK IKKFYNKSTF
Cel-PGP-12,CE03260                  LAGVGKAGSI AEESLMGVRT VQAFNGQEEM VEKYTVELGK GKSFAIQKGL
Cel-PGP-13,CE53174                  LEKVGEAGAI AEECLMGVRT IQAFNGQEEM VAKYEKQLNS GKKHAIWGGF
Cel-PGP-14,CE03262                  LIGVGKAGSI AEESLMGVRT VQAFNGQEEM VGRYEAELEK GRKFAVWKGF
Hco-PGP-1,HCON_00098130-00001       AIKYAKAGRM VEQTVSSIRT VCSLNGLRYE IERYKVALLE ARRAGILKSL
Hco-PGP-2,HCON_00004450-00001       QETYAVAGAI AEETFSSIRT VHSLCGHKRE LTRFEAALEK GRQTGLVKYF
Hco-PGP-3,HCON_00042800-00001       NYAYSSAAAL ANEVIAGIRT VMAFNAQPFE IHRYEKELKE ARKLGIRKAF
Hco-PGP-9.1,HCON_00130050-00001     AKKYAVAGGI AEEVLTSIRT VIAFNGQPYE CERYQKALED GKSTGIKKSF
Hco-PGP-9.2,HCON_00130060-00001     AKKYAVAGGI AEEVLTSMRT VMAFNGQPYE CERYKQALED GKSTGIKKSL
Hco-PGP-10,HCON_00168800-00001      VHLQNKISNL VNETFSCIRT VISFAAQKQT ITKFERLSME NNKLTESRLR
Hco-PGP-11,HCON_00162780-00001      MSVSGEAGAI AEEAIMNVKT VAACNGQKHM VKKYDEQLRR GVSFAIRYSF
Hco-PGP-13,HCON_00041390-00001      LVGVGKAGSI AEESLMGVRT VQAFNGQQEM VDRYSTELAR GKSIAIWKGF
Hco-PGP-16,HCON_00035895-00001      QEFYVRAGST AEEVVNGIRT VVAFNGQEKE IKRYSDYLEQ GMRQGMVKAF
Hsa-ABCB1,NP_001335874.1            LLAYAKAGAV AEEVLAAIRT VIAFGGQKKE LERYNKNLEE AKRIGIKKAI
Mmu-ABCB1,NP_035205.1               LQAYAKAGAV AEEVLAAIRT VIAFGGQQKE LERYNKNLEE AKNVGIKKAI
```

```
                                  401
Cel-PGP-1,CE11932                 FLGISFGAMQ A-SNFISFAL AFYIGVGWVH D-GSLNFGDM LTTFSSVMMG
Cel-PGP-2,CE41207                 YMGIGVGFSN L-CMYSSYAL AFWYGSTLII NDPTFDRGLI FTVFFAVLSG
Cel-PGP-3,CE03818                 ILAICTAFPL M-LMFTCMAV AFWYGATLAA A-GAVSSGAV FAVFWAVLIG
Cel-PGP-4,CE44238                 VLALCSAMPL F-LMFVLMAG AFWYGAILTS Y-GVATSGTT FGVFWAVILG
Cel-PGP-5,CE43003                 VFSLSRSADY F-FTNALNFV ILYFGANMIY E-GTIEPGVV VRILYYILFG
Cel-PGP-6,CE40818                 MYSISRGVTY F-FCNSLNTV VLYVGATMIY S-GTLETAVV VRLFHYMLFG
Cel-PGP-7,CE53605                 MYSISRGFTY F-FCNSLNTV ILYVGANMIY S-GSLEPAVV VRIFHYMMFG
Cel-PGP-8,CE31624                 FLATSRGLSH F-FCNALNGT ILYVGADLIY N-KTMNTVAI VTLFHYMLFS
Cel-PGP-9,CE15714                 LIGAGLASFF V-IIYASYCL AFWVGTNFVY S-GRLESGTV LTVFFSVMMG
Cel-PGP-10,CE40807                SSVVFDSLTQ ILLTELIFTG ALCYGIWRVA D---NSPGRL CALAINMLYM
Cel-PGP-11,CE53819                WAGFFDGLAL F-VIYFITGI SLFFGCRLYF NQEIGKAGDV ILIVNTICVT
Cel-PGP-12,CE03260                WGGVFGGIFL L-FLFSYFGG GIYYGGQLLR WKIIETPGDV FIVVISMLIG
Cel-PGP-13,CE53174                WSGFFGGIFF F-WLMAFMGC GILYGGYLLK VGIIKSPGDV FIIVVAMLLG
Cel-PGP-14,CE03262                WSGFFGGLFF F-WLFSFLGC GMLYGAYLLK VGIITTPGDV FIVVMSMLLG
Hco-PGP-1,HCON_00098130-00001     FVGLSFALMG L-TNFSSFAL AFYIGITWTV D-GQLELKDL MTTFFSVMMG
Hco-PGP-2,HCON_00004450-00001     YMGVGVGFGQ M-CTYVSYAL AFWYGSVLII NDPALDRGRI FTVFFAVMSG
Hco-PGP-3,HCON_00042800-00001     ILAMFSGLPL F-LMFAAMAF SFWYGSTLVI A-GIVSPGTI FAVFWSVFLG
Hco-PGP-9.1,HCON_00130050-00001   YIGVGLGITF L-IMFSSYCL AFWVGTDFVF K-GQMNGGTV MTVFFSVMMG
Hco-PGP-9.2,HCON_00130060-00001   YVGIGFAITF L-ILFSSYCL AFWVGTDFVY N-NRMQGGTV MTVFFAVMMG
Hco-PGP-10,HCON_00168800-00001    SSTVYDSLTQ ILLTELIFTA ALCYGIWRVA D---HSPGRL AALAINMLYM
Hco-PGP-11,HCON_00162780-00001    INGFCEGFMF F-QLYIFYAA AFLYGIPSYY NGITAEPGTI FIVASTVLLG
Hco-PGP-13,HCON_00041390-00001    WSGLLGGLFF F-ALFSFLGC GMLYGGYLLK VNIIKTPGDV FIVVMSLLLG
Hco-PGP-16,HCON_00035895-00001    LTSFGTAFIM G-SLFVSMGI SFWYGTKLVI S-GAITPGTV FSVFWAVIGG
Hsa-ABCB1,NP_001335874.1          TANISIGAAF L-LIYASYAL AFWYGTTLVL S-GEYSIGQV LTVFFSVLIG
Mmu-ABCB1,NP_035205.1             TASISIGIAY L-LVYASYAL AFWYGTSLVL S-NEYSIGEV LTVFFSILLG

                                  451
Cel-PGP-1,CE11932                 SMALGLAGPQ LAVLGTAQGA ASGIYEVLDR K---PVIDSS SKAGR-K---
Cel-PGP-2,CE41207                 STSLGGALPH LASFGTARGA ASTVLRVINS H---PKIDPY SLEGI-L---
Cel-PGP-3,CE03818                 TRRLGEAAPH LGAITGARLA IHDIFKVIDH E---PEIKCT SSEGK-I---
Cel-PGP-4,CE44238                 TRRLGEAAPH MGAITGARLA VNDIFKVIDH E---PEINCT KQEGR-R---
Cel-PGP-5,CE43003                 SYCLGEAILH ISRLASAIPL TVPIADILLD SDATADE-FF --SEE-I---
Cel-PGP-6,CE40818                 AFCLSEALPH ISRLAGAISS TAPIAEMLIK DDNVIEKDET --DYD-V---
Cel-PGP-7,CE53605                 AFCLSEALPH ISRLAGAISS TAPIAEMLIK EDNVIEKDET --DYD-V---
Cel-PGP-8,CE31624                 AFSLGEAFLH ISYLLNAINS ATPIFDVLTS DDDMIENNQD NEQTD-S---
Cel-PGP-9,CE15714                 SMALGQAGQQ FATIGTALGA AASLYEVIDR I---PEIDAY STEGQ-T---
Cel-PGP-10,CE40807                CVTSISIGFH MNGATRAKDN AKEICEVLNE Q---PKIEVE LASIDNNIDY
Cel-PGP-11,CE53819                GYFLGLLGPH MSSLQQAATS FLLLYKTIES A---PKSE-- GKEGEIK---
Cel-PGP-12,CE03260                AYFLGLISPH LMVLLNARVA AATIYEIIDR T---PDIDVY STEGQ-K---
Cel-PGP-13,CE53174                AYFLGLISPH LMVLLNARVA AASIYKTIDR V---PKIDPY SRHGK-K---
Cel-PGP-14,CE03262                AYFLGLISPH MMVLLNARVS AASIYQTIDR V---PKIDPY SKAGK-R---
Hco-PGP-1,HCON_00098130-00001     SLALGQAGPQ FAVLGAAQGA AASIYEVLDR E---PEIDST SPEGR-R---
Hco-PGP-2,HCON_00004450-00001     SAALGTCLPH LNTISIARGA VRSVLSVINS R---PKIDPY SLDGI-V---
Hco-PGP-3,HCON_00042800-00001     TRRLSDVAPQ LGAFLGAKIA AADIFAVIDR V---PEIDPM SNDGL-T---
Hco-PGP-9.1,HCON_00130050-00001   SMALGQAGPQ FAVLGTAMGA AGSLYQIIDR E---PEIDSY STDGV-K---
Hco-PGP-9.2,HCON_00130060-00001   SMALGQAGPQ FAVLGTAMGA AGSLYQIIDR E---PEIDSY SQEGV-R---
Hco-PGP-10,HCON_00168800-00001    CVTSISIGFH INGASTAFQS ASQLRAILDE C---PRIESD YGFAGDNPT-
Hco-PGP-11,HCON_00162780-00001    SYFFGLLGPH MMAIMKARIA AAIIYETIDQ A---QDIV-- SKEGK-E---
Hco-PGP-13,HCON_00041390-00001    AYFLGLISPH LMVLLNARVA AATIYQTIDR V---PKIDVY SEKGR-K---
Hco-PGP-16,HCON_00035895-00001    AFAVGQAAPQ IGVLISSMTA AAPIFSIIDR K---PPIDSL SQEGR-V---
Hsa-ABCB1,NP_001335874.1          AFSVGQASPS IEAFANARGA AYEIFKIIDN K---PSIDSY SKSGH-K---
Mmu-ABCB1,NP_035205.1             TFSIGHLAPN IEAFANARGA AFEIFKIIDN E---PSIDSF STKGY-K---
```

```
                                      501
Cel-PGP-1,CE11932                     ---------- ---DMKIKGD ITVENVHFTY PSRPDVPILR GMNLRVNAGQ
Cel-PGP-2,CE41207                     ---------- ---VDNMKGD ISFKDVHFRY PSRKDIHVLK GISLELKAGD
Cel-PGP-3,CE03818                     ---------- ---PEKIQGK LTFDGIEFTY PTRPELKILK GVSFEVNPGE
Cel-PGP-4,CE44238                     ---------- ---PDKVNGK LVFDNIQFTY PTRPDVKILK GVSFEVNPGE
Cel-PGP-5,CE43003                     ---------- ---KDTFQGI ISFKNVLFSY PTRPDVPVLK EISFNVQGGE
Cel-PGP-6,CE40818                     ---------- ---EVEVNGN ISFKNVKFSY PTRPDAQVLK GISFDVQNGE
Cel-PGP-7,CE53605                     ---------- ---EVEVNGN ISFKNVKFSY PTRPDAQVLK GISFDVQNGE
Cel-PGP-8,CE31624                     ---------- ---NKTIQGM LSFNNVKFAY PSRPDVDILR GISFDVKQGE
Cel-PGP-9,CE15714                     ---------- ---PSKISGR ISVNKVEFTY PTRADVKILK GVSLDAQPGQ
Cel-PGP-10,CE40807                    VPVTRCKYRR QSMKFMGKGA LHFRDIHFSY PSRKETEVLR GISFKVEAGE
Cel-PGP-11,CE53819                    ---------- ---ISSTRGN IEFRDVRFKY FTR-DNEVLQ GLSLQVLPGQ
Cel-PGP-12,CE03260                    ---------- ---MTNVVGK VVFEEVHFRY PTRKKVKVLN GLNLTVKPGT
Cel-PGP-13,CE53174                    ---------- ---IEKVVGK VTFENVHFRY PTRKEAKVLN GLNLTVEPGT
Cel-PGP-14,CE03262                    ---------- ---LQNVVGR VKFENVHFRY PSRKDAKILN GLNLVVEPGT
Hco-PGP-1,HCON_00098130-00001         ---------- ---DVDIKGN IVVKNVVFNY PSRPDVPVLK NLSLTVNAGE
Hco-PGP-2,HCON_00004450-00001         ---------- ---LNNMRGS IRFKNVHFSY PSRRTLQILK GVSLQVSAGQ
Hco-PGP-3,HCON_00042800-00001         ---------- ---PEEFVGR LTFSNIHFTY PSRPTVKILD GVSFEVNPGE
Hco-PGP-9.1,HCON_00130050-00001       ---------- ---PSNLKGK VTVSNLKFTY PTRPDVPILK GVSFEANPGE
Hco-PGP-9.2,HCON_00130060-00001       ---------- ---PSTLTGK LTISNLKFTY PTRPDVPILK GVSFEANPGE
Hco-PGP-10,HCON_00168800-00001        RCLPAPKYRR QSMKYMGKGA IHFRDIHFSY PSRPDVEVLK GVTFHVEAGE
Hco-PGP-11,HCON_00162780-00001        ---------- ---LRACKGR LEFRDVHFKY PTR-ETPILQ GLSWVAEPGE
Hco-PGP-13,HCON_00041390-00001        ---------- ---PDRIHGR VVFENVHFRY PSRKDVKVLN GLNLVIEPGQ
Hco-PGP-16,HCON_00035895-00001        ---------- ---LEDVRGR ISISNVHFSY PSRPEVEVLK GVSLNVESGQ
Hsa-ABCB1,NP_001335874.1              ---------- ---PDNIKGN LEFRNVHFSY PSRKEVKILK GLNLKVQSGQ
Mmu-ABCB1,NP_035205.1                 ---------- ---PDSIMGN LEFKNVHFNY PSRSEVQILK GLNLKVKSGQ

                                      551
Cel-PGP-1,CE11932                     TVALVGSSGC GKSTIISLLL RYYDVLKGKI TIDGVDVRDI NLEFLRKNVA
Cel-PGP-2,CE41207                     KIALVGSSGC GKSTIVNLLQ RFYDPTKGRV LIDGVDLREV NVHSLREQIG
Cel-PGP-3,CE03818                     TVALVGHSGC GKSTSIGLLM RFYNQCAGMI KLDGIPIQEY NIRWLRSTIG
Cel-PGP-4,CE44238                     TIALVGHSGC GKSTSIGLLM RFYNQCAGSI KLDGIPIEDY NIQWLRSTIG
Cel-PGP-5,CE43003                     CIALVGASGS GKSTVIQLLL HYYNIDSGRI SIDGNDIYNI NIKQLRQAMG
Cel-PGP-6,CE40818                     CIALVGASGS GKSTVVQLLL HYYNIDSGNI FIDGMDLNDM NIKRLRRVIG
Cel-PGP-7,CE53605                     CIALVGASGS GKSTVVQLLL HYYNIDSGNI FIDGMDLNDM NIKRLRRVIG
Cel-PGP-8,CE31624                     CIALVGASGS GKSTIVQLLL HFYNIQSGTI KIGDSHLHDI NLKQLRNAIG
Cel-PGP-9,CE15714                     TVALVGSSGC GKSTIIQLLQ RFYNPDAGQI LIDDIPIEDF NIKYLRQLVG
Cel-PGP-10,CE40807                    KIAIVGSSGS GKSTLTALLL RFYDPTKGAI LLDGENIKTM CPDDLRGQCS
Cel-PGP-11,CE53819                    TVALVGTSGC GKSTSIGLLT KLYRASEGEI LIDGKNIDML DAKSLRQQIG
Cel-PGP-12,CE03260                    SVALVGHSGC GKSTSVGLLT RLYEPEGGKV MIDGQDVRSL NIDWLRKTVG
Cel-PGP-13,CE53174                    SVALVGHSGC GKSTSVGLLT RLYEPEQGSV QIDGVDVRDL NLEWLRNVVG
Cel-PGP-14,CE03262                    SVALVGHSGC GKSTSVGLLT RLYEPEAGNV TIDGTDVREL NIEWLRNTVG
Hco-PGP-1,HCON_00098130-00001         TVALVGSSGC GKSTIVSLLL RYYNVLKGEI IIDGVPISDI NIQYLRNRIA
Hco-PGP-2,HCON_00004450-00001         KIALVGSSGC GKSTIVNLLL RFYDPTRGKV TIDDIDVCDL NVQKLREQIG
Hco-PGP-3,HCON_00042800-00001         TVALVGHSGC GKSTIISLLL RFYEQSAGMV ALDGIPLRDY NVKWWRSVVG
Hco-PGP-9.1,HCON_00130050-00001       TIALVGSSGC GKSTIIQLLL RYYNPEDGKI TIDGVEIDKI NIEFLRNYVG
Hco-PGP-9.2,HCON_00130060-00001       TIALVGPSGC GKSTIVQLLL RYYNPDDGKI TIDGVEIDKL NIEFLRNYVG
Hco-PGP-10,HCON_00168800-00001        KIALVGSSGS GKSTLTALLL RFYDPDSGSI LLDGDNLKTL CPDDLRGMCS
Hco-PGP-11,HCON_00162780-00001        TIAFVGKSGC GKSTSIGLLT RLYDCDKGSA LLDGQEIRSI KTSDLRKMIG
Hco-PGP-13,HCON_00041390-00001        TVALVGHSGC GKSTSVGLLT RLYEPESGRV TIDGEDVREL NIDWLRNAVG
Hco-PGP-16,HCON_00035895-00001        HIALVGHSGC GKSTLFGLLL RFYEQQSGKV SIDDVLVSDI NVEHLRNIIG
Hsa-ABCB1,NP_001335874.1              TVALVGNSGC GKSTTVQLMQ RLYDPTEGMV SVDGQDIRTI NVRFLREIIG
Mmu-ABCB1,NP_035205.1                 TVALVGNSGC GKSTTVQLMQ RLYDPLEGVV SIDGQDIRTI NVRYLREIIG
```

```
                                          601
Cel-PGP-1,CE11932                         VVSQEPALFN CTIEENISLG KEGITREEMV AACKMANAEK FIKTLPNGYN
Cel-PGP-2,CE41207                         IVSQEPVLFD GTIYENIKMG NEHATHDQVV EACKMANAND FIKRLPDGYG
Cel-PGP-3,CE03818                         IVQQEPIIFV ATVAENIRMG DVLITDQDIE EACKMANAHE FICKLSDRYD
Cel-PGP-4,CE44238                         IVQQEPIIFL ATVAENVRMG DDSITDKDIE NACRQANAHD FIGKLSEGYN
Cel-PGP-5,CE43003                         VVFQEPVLFN TSIEENIRFG KPDATEQEII DALKNANAFD FVCNFPDGIK
Cel-PGP-6,CE40818                         VVSQEPVLFN TTIEENIRFG NPNVSLPEIY GALRKANAYD FVCSFPKGIK
Cel-PGP-7,CE53605                         VVSQEPVLFN TTIEENIRFG NPNVSLPEIY GALRKANAYD FVCSFPKGIK
Cel-PGP-8,CE31624                         VVSQEPVLFN TTIEENIRFG NPNATSSEIY EALRKANAYD FVCNIKDGLK
Cel-PGP-9,CE15714                         VVSQEPNLFN TSIEQNIRYG RSDVSDEDIA RALKEANAAD FIKTFPEGLN
Cel-PGP-10,CE40807                        LVSQEPVLFD GTISDNIRYG RLDATQQEIN DAARKVGAWK FINSLPDGMQ
Cel-PGP-11,CE53819                        IVQQEPKLFD GTIMENIKLG R-NVDEETIK TAADIANASS FIEKLENGYE
Cel-PGP-12,CE03260                        IVQQEPILFN DTIHNNLLIG NPSATREDMI RVCKMANAHD FIQKMPNGYE
Cel-PGP-13,CE53174                        IVQQEPILFN DTIHNNLLFG NPDATRETMI RVCKMANAHD FIKKMPKGYD
Cel-PGP-14,CE03262                        IVQQEPILFN DTIHNNLLIG NPGSTRETMI EVCKMANAHD FIEKMPKGYD
Hco-PGP-1,HCON_00098130-00001             VVSQEPILFN CTIEENIRMG RSDLTYQQVV DACRMANAET FINGLPQGLQ
Hco-PGP-2,HCON_00004450-00001             VVSQEPVLFD GTLFENIKMG YEQATMEEVQ EACRVANAAD FIKRLPEGYG
Hco-PGP-3,HCON_00042800-00001             VVQQEPVIFC ATVAENVRMG DDSLTDEDVE EACKLANALG FINKLSEGFN
Hco-PGP-9.1,HCON_00130050-00001           VVSQEPMLFN TTIEQNIRYG RENVTDAEIT AALRKANAYN FVQSFPDGIY
Hco-PGP-9.2,HCON_00130060-00001           VVSQEPMLFN TTIEQNIRYG RENVTDAEIT AALRKANAYD FVQSFPDGIY
Hco-PGP-10,HCON_00168800-00001            LVSQEPVLFD GTISDNIRYG RLDATQQEIN DAARKVGAWQ FISSLPEGMQ
Hco-PGP-11,HCON_00162780-00001            IVQQEPCLFN GTIRENIVLG R-SITDEQAE DAARIANAHD FIMKLDKGYD
Hco-PGP-13,HCON_00041390-00001            IVQQEPCLFN DTVAGNLRMG NPTMSLEQMV YVCKMANAHD FIGKLPNAYE
Hco-PGP-16,HCON_00035895-00001            VVSQEPALFA DTVENNIRLG RADISQKEME DCCKMANAHE FIMNLSQGYQ
Hsa-ABCB1,NP_001335874.1                  VVSQEPVLFA TTIAENIRYG RENVTMDEIE KAVKEANAYD FIMKLPHKFD
Mmu-ABCB1,NP_035205.1                     VVSQEPVLFA TTIAENIRYG REDVTMDEIE KAVKEANAYD FIMKLPHQFD

                                          651
Cel-PGP-1,CE11932                         TLVGDRGTQL SGGQKQRIAI ARALVRNPKI LLLDEATSAL DAESEGIVQQ
Cel-PGP-2,CE41207                         TRVGEKGVQL SGGQKQRIAI ARALVKNPKI LLLDEATSAL DTEAEREVQG
Cel-PGP-3,CE03818                         TVIGAGAVQL SGGQKQRVAI ARAIVRKPQI LLLDEATSAL DTESERMVQT
Cel-PGP-4,CE44238                         TVIGAGAVQL SGGQKQRVAI ARAIVRKPQI LLLDEATSAL DTESERMVQT
Cel-PGP-5,CE43003                         TIVGERGAQL SGGQKQRIAI ARTLVRNPRI LLLDEATSAL DNESEFIVQE
Cel-PGP-6,CE40818                         TIVGERGTQL SGGQKQRIAI ARTLVRNPKI LLLDEATSAL DNESEQVVQK
Cel-PGP-7,CE53605                         TIVGERGTQL SGGQKQRIAI ARTLVRNPKI LLLDEATSAL DNESEQVVQK
Cel-PGP-8,CE31624                         TIVGERGAQL SGGQKQRIAI ARVLVKNPAI LLLDEATSAL DSASERAVQL
Cel-PGP-9,CE15714                         TLVGDRGVQM SGGQKQRIAI ARALVRNPKI LLLDEATSAL DAESESIVQS
Cel-PGP-10,CE40807                        TRVGERGHQL SGGQKQRVAI ARAVIRKPIV LIFDEATSAL DNIHEEEVNA
Cel-PGP-11,CE53819                        TRLGPGGVQL SGGQKQRICI ARALVTSPSI LLLDEATSAL DSHNEHIVNK
Cel-PGP-12,CE03260                        TMIGDGSVQL SGGQKQRVAI ARTLIRDPKV LLLDEATSAL DAQSESVVQS
Cel-PGP-13,CE53174                        TQIGDGGVQL SGGQKQRVAI ARTLIRDPKV LLLDEATSAL DAQSESVVQS
Cel-PGP-14,CE03262                        TLIGDGGVQL SGGQKQRVAI ARTLIRDPKV LLLDEATSAL DAQSESIVQS
Hco-PGP-1,HCON_00098130-00001             TNVGDRGTQL SGGQKQRIAI ARALVRDPKI LLLDEATSAL DAESEAVVQR
Hco-PGP-2,HCON_00004450-00001             TRVGERGVQL SGGQKQRIAI ARAIIKNPRI LLLDEATSAL DTEAESIVQE
Hco-PGP-3,HCON_00042800-00001             TVIGEGAVQL SGGQKQRIAI ARALVRNPQI LLLDEATSAL DTESERAVQE
Hco-PGP-9.1,HCON_00130050-00001           TNVGDRGTQM SGGQKQRIAI ARALVRDPKI LLLDEATSAL DAESEHIVQQ
Hco-PGP-9.2,HCON_00130060-00001           TNVGDRGTQM SGGQKQRIAI ARALVRDPKI LLLDEATSAL DAESEHVVQQ
Hco-PGP-10,HCON_00168800-00001            TRVGDRGLQL SGGQKQRVAI ARAVIRKPTV MLFDEATSAL DNLHEEEVQH
Hco-PGP-11,HCON_00162780-00001            TIIGSGGVSL SGGQKQRIAI ARAVATQPKI LLLDEATSAL DSESENVVQL
Hco-PGP-13,HCON_00041390-00001            TYIGDGGVQL SGGQKQRIAI ARTLARDPKV LLLDEATSAL DAQSESIVQS
Hco-PGP-16,HCON_00035895-00001            TKIGEGGVQL SGGQKQRVAI ARALVRNPRI LLLDEATSAL DAESESIVQQ
Hsa-ABCB1,NP_001335874.1                  TLVGERGAQL SGGQKQRIAI ARALVRNPKI LLLDEATSAL DTESEAVVQV
Mmu-ABCB1,NP_035205.1                     TLVGERGAQL SGGQKQRIAI ARALVRNPKI LLLDEATSAL DTESEAVVQA
```

```
                                    701
Cel-PGP-1,CE11932                   ALDKAAKGRT TIIIAHRLST IRNADLIISC KNGQVVEVGD HRALMA-QQG
Cel-PGP-2,CE41207                   ALDQAQAGRT TIIVAHRLST IRNVDRIFVF KAGNIVESGS HEELMS-KQG
Cel-PGP-3,CE03818                   ALDKASEGRT TLCIAHRLST IRNASKILVF DQGLIAERGT HDELISKDDG
Cel-PGP-4,CE44238                   ALDKASEGRT TLCIAHRLST IRNASKILVF DQGLIPERGI HDQLIR-QNG
Cel-PGP-5,CE43003                   ALQKASIGRT TIVVAHRLST IRNANKIIVM EKGEIVEVGD HKQLIA-MNG
Cel-PGP-6,CE40818                   ALENASQGRT TIVVAHRLST IRNASKIIVM QKGEIVEVGN HDELIA-KRG
Cel-PGP-7,CE53605                   ALENASQGRT TIVVAHRLST IRNASKIIVM QKGEIVEVGN HDELIA-KRG
Cel-PGP-8,CE31624                   ALKKASEGRT TIIIAHRLST IRHCDKIMVM SNGKIAEVGS HEELIS-MDR
Cel-PGP-9,CE15714                   ALENASRGRT TIVIAHRLST VRNADKIIVM KAGQVMEVGT HETLIE-QKG
Cel-PGP-10,CE40807                  AIDLASEGLT TITIAHRLST IKNSDRIIVL HEGKIVEEGV PDELIADANG
Cel-PGP-11,CE53819                  ALTKASEGRT TIIIAHRLSS LKSVDRIYVL DQGKTKEIGT HDELIL-LGG
Cel-PGP-12,CE03260                  ALNNAAKGRT TIMIAHRLST IREADKIVFF ENGVIVESGN HEELVA-LGG
Cel-PGP-13,CE53174                  ALNNASKGRT TIMIAHRLST IREADKIVFF EKGVIVEAGN HEELVN-LGG
Cel-PGP-14,CE03262                  ALNNASKGRT TIMIAHRLST IREADKIVFF EKGVIVEAGN HEELVR-LGG
Hco-PGP-1,HCON_00098130-00001       ALEKASIGRT TIIIAHRLST IKNAHKIIAI KAGEVEEVGT HEELLA-NKG
Hco-PGP-2,HCON_00004450-00001       ALEKAQKGRT TVIVAHRLST IRNVDQIFVF KNGTIVEQGT HAELMN-KRG
Hco-PGP-3,HCON_00042800-00001       ALDKARENRT TLCIAHRLST IRDSDKIIVF DEGHIVEQGT HDELMSIEDG
Hco-PGP-9.1,HCON_00130050-00001     ALENASKGRT TIVVAHRLST IRNADKIVAM KNGEVMEVGT HDELIA-RKG
Hco-PGP-9.2,HCON_00130060-00001     ALENASKGRT TIVVAHRLST IRNADRIIAI KDGEVMEVGT HDELMA-RKE
Hco-PGP-10,HCON_00168800-00001      AIDLASEGLT TITIAHRLST VKNCDRIIVM DEGRIVEEGP LDELLAKEDS
Hco-PGP-11,HCON_00162780-00001      ALNRASRGRT TIVIAHRLST LKDVQRIYAI QDGKVVEAGT HFELLE-KGG
Hco-PGP-13,HCON_00041390-00001      ALNNASRGRT TIVIAHRLST IRDANKIVFF EKGQIVEQGT HQELVA-SRG
Hco-PGP-16,HCON_00035895-00001      ALENAQSGRT TISIAHRLST IKNVDRIYVF NNGRIVEDGN HDELMK-MNG
Hsa-ABCB1,NP_001335874.1            ALDKARKGRT TIVIAHRLST VRNADVIAGF DDGVIVEKGN HDELMK-EKG
Mmu-ABCB1,NP_035205.1               ALDKAREGRT TIVIAHRLST VRNADVIAGF DGGVIVEQGN HDELMR-EKG

                                    751
Cel-PGP-1,CE11932                   LYYDLVTAQT FTDAVDSAAE GKFSREN--- ---------- SV----ARQT
Cel-PGP-2,CE41207                   IFYDMTQAQV VRQQQQEAGK DIEDTIS--- ---------- ----------
Cel-PGP-3,CE03818                   IYASMVKAQE IERAKEDTTL DDEEDE---- -KTHRSFHRD SV----TSDE
Cel-PGP-4,CE44238                   IYANMVRAQE IEKAKDDTTQ DDDELVE--- -EDNYSISRR LS----TSEE
Cel-PGP-5,CE43003                   VYNNLVQTQL MSTNYEKMNE NEERVTR--- -QSSHS---- ----------
Cel-PGP-6,CE40818                   VYNDLVQAQL LESHDDHEEL P-PLAAR--- -QLSQEL--- ----------
Cel-PGP-7,CE53605                   VYNDLVQAQL LESHDDHEEL P-PLAAR--- -QLSQEL--- ----------
Cel-PGP-8,CE31624                   EYSNLVRAQF FDSQSVEEDI NGQGAEE--- -VIQKT---- ----------
Cel-PGP-9,CE15714                   LYHELVHAQV FADVDDKPKK KEAERRM--- ---------- SR-------Q
Cel-PGP-10,CE40807                  IFHKMYNDQR LDQLTQQHQQ LHQQQAKPPS LQQQMSFSPP PNMDPDQQRR
Cel-PGP-11,CE53819                  IYARLAKSQE VEQSSKKDWE REELRRA--- -EK-MKKKGR TV-EIIE---
Cel-PGP-12,CE03260                  RYAKLVEAQK FKESDDIEDN GDEHEEE--- -TSTVGRHDR LS----SRQV
Cel-PGP-13,CE53174                  RYFDLVKAQA FKQDPDEIAL EKEEEDQ--- -FDEFDKPTV FN-----RKV
Cel-PGP-14,CE03262                  RYFDLVKAQQ FKADPEATEE FEEEEID--- -LDDT----- -------SRS
Hco-PGP-1,HCON_00098130-00001       LYYDLVHAQT FTDAVDDVNA NEAVEKF--- ---------- SI----GSQK
Hco-PGP-2,HCON_00004450-00001       VFFEMTQAQV LRQEKEEEVL ENTEPVA--- -KCQEVSLPA PDVTILTPHE
Hco-PGP-3,HCON_00042800-00001       VYRSMVKAQA IEKGEEDTTL DDVDPTE--- -I-RRGVSR- -V----VSED
Hco-PGP-9.1,HCON_00130050-00001     LYHELVNAQV FADVDDKSGE PGDRRRT--- ---------- MS----SSRS
Hco-PGP-9.2,HCON_00130060-00001     LYHELVNSQV FVDVDGDGEE AGAR-RT--- ---------- PP----LSRS
Hco-PGP-10,HCON_00168800-00001      RFRRLYNDQR MDILAQSQPQ SKTSLV--PA ASFALGQHYR PQIHPLQHRR
Hco-PGP-11,HCON_00162780-00001      LYSALAAAQE VGVNIADIST RTR-KYS--- -DS-IDSHAY SA--------
Hco-PGP-13,HCON_00041390-00001      RYYELVKAQQ FEPEAEEVE- --EEEVD--- -LGDNDGGSL LS-----GRS
Hco-PGP-16,HCON_00035895-00001      LYSELVRAQE IEQLEKSGSD DETAEH---- ---NVTLM-- ----------
Hsa-ABCB1,NP_001335874.1            IYFKLVTMQT AGNEVELENA ADESKSE--- ---------- ID----ALEM
Mmu-ABCB1,NP_035205.1               IYFKLVMTQT RGNEIEPGNN AYGSQSD--- ---------- TD----ASEL
```

```
                                   801
Cel-PGP-1,CE11932                  S-EHEG---L -----S--RQ --ASEMDD-- -----IMNRV RSSTIGSITN
Cel-PGP-2,CE41207                  ---------- ---------- ---------- -----ESAHS HLSRKSS---
Cel-PGP-3,CE03818                  E----R---E -----L--Q- -----QSL-- -----ARDST RLRQ------
Cel-PGP-4,CE44238                  E----L---R -----K--S- -----KSL-- -----LRDST RFSQ------
Cel-PGP-5,CE43003                  ---------- ---------- ------DF-- ---------- -PSN------
Cel-PGP-6,CE40818                  ---------- -----S--PL -----HSY-- ---------- -AIQ------
Cel-PGP-7,CE53605                  ---------- -----S--PL -----HSY-- ---------- -AIQ------
Cel-PGP-8,CE31624                  ---------- -----P--P- ---------- ---------- ----------
Cel-PGP-9,CE15714                  T-SQRK---G -----S--V- ------NF-- -----KTQES QVD------E
Cel-PGP-10,CE40807                 AWARSSLGGN -KKLGKSYSV NSMEKEKLKL PVMSKKKS-E RLDYKY----
Cel-PGP-11,CE53819                 -----P---N -----S--TI LQEHEHNFVG SVITEN---- ----------
Cel-PGP-12,CE03260                 S-FHKS---C -----E--SL AS---ADL-- -----E--IG YASTFNT---
Cel-PGP-13,CE53174                 S-VRNS---R -----S--SG RSGS-EEF-- -----RRGSL ANHSFDR---
Cel-PGP-14,CE03262                 S-RRSS---M -----T--SA RSGS-EAF-- -----RRGNS LNDSFSG---
Hco-PGP-1,HCON_00098130-00001      K-A-SL---L -----S--RE TSVQSAND-- -----LHLRV RTATIMTMDE
Hco-PGP-2,HCON_00004450-00001      E-QPEL---P -----S--PP DSDAESDVVS PDIALPHLSS LRSRKES---
Hco-PGP-3,HCON_00042800-00001      D----S---Q -----S--R- -----ADR-- -----ARESA RLRQ------
Hco-PGP-9.1,HCON_00130050-00001    R-SPSL---A -----S--P- ------EY-- -----KRLKS QMSTENA-A-
Hco-PGP-9.2,HCON_00130060-00001    K-SPSN---N -----S--A- ------DF-- -----MGLGS QLSTNDD-KR
Hco-PGP-10,HCON_00168800-00001     SWNHSSHGGK KKKLGKSYSV LSHD--KERL PVMSKKRS-Q RLDYKC----
Hco-PGP-11,HCON_00162780-00001     ---------- ---------- -ALVRHDLRG SGMTRRSVS- ----------
Hco-PGP-13,HCON_00041390-00001     ----TL---T -----Q--SK RSGS-EAF-- -----VRGQA LNDSFGR---
Hco-PGP-16,HCON_00035895-00001     ---------- ---------- ---------- -----RRRSK RLSR------
Hsa-ABCB1,NP_001335874.1           S-SNDS---R -----S--S- -------L-- -----IRK-- -RSTRRS---
Mmu-ABCB1,NP_035205.1              T-SEES---K -----S--P- -------L-- -----IRR-- --SIYRS---

                                   851
Cel-PGP-1,CE11932                  GPVIDEKEER IG--KDALSR LKQELEENNA QKTNLFEILY HARPHALSLF
Cel-PGP-2,CE41207                  -----TRSAI SI--ATSIHQ LAEEVEECKA PPTSMFKIFK FNGDKVGWFI
Cel-PGP-3,CE03818                  --SMISTTTQ VP--EWEIEN AREEMIEEGA MEASLFDIFK YASPEMRNII
Cel-PGP-4,CE44238                  --SMLSVTSQ VP--DWEMES AREEMIEEGA MEASMMDIFR FAKPEKMNIV
Cel-PGP-5,CE43003                  --EISHQKID QE--DDYVKK LIAEIKEEGA KKSNICEIIK YCRSEYCILF
Cel-PGP-6,CE40818                  --RSTSNDAG VH--DDDMER ILDELSKEGA KKSNLREIVK QCRPDYCFLF
Cel-PGP-7,CE53605                  --RSTSNDAG VH--DDDMER ILDELSKEGA KKSNLREIVK QCRPDYCFLF
Cel-PGP-8,CE31624                  --------NL ND--GEPLEE LLKETSSDIE IKSNIWEVLR ECRPDAFLLS
Cel-PGP-9,CE15714                  KPG-APPAPE AA--EKEIKR LKKELEEEGA VKANLFKILR YARPEWIYIF
Cel-PGP-10,CE40807                 --SKVHSMDV KQHT--DSMH DDDDLPGKET NFTVLKDLIY SYRTGLPLLA
Cel-PGP-11,CE53819                 ---------- --------EE QKISF-SGIS KLFNYLPKH- ---RKTLI--
Cel-PGP-12,CE03260                 FTLKTAQEEI EN--EDFAEE VQRVMEEDGV INSGYLDIFK NAQGNYLYLS
Cel-PGP-13,CE53174                 FRKASHIPSA ED--EAFALR VKETMEKDGE ITAGFLDIFK NAQGNYTYML
Cel-PGP-14,CE03262                 SKRSA-QADA EN--SAFAAN EAAIMAEDGQ ITAGYLDIFK NAKGNYLYMF
Hco-PGP-1,HCON_00098130-00001      QQM---DETK EK--KDDITR LRKELKAEGV KKTNLLEILA YASPHWKMLT
Hco-PGP-2,HCON_00004450-00001      -----TRSAI SA--VPSVRS MQIEMEDLRA KPTPMSKIFY FNRDKWGYFI
Hco-PGP-3,HCON_00042800-00001      --SMVSASTQ EP--EWEIES ARDNLIEEGG MEASLLDILC YAKPELPMAG
Hco-PGP-9.1,HCON_00130050-00001    GGG-AQNDPV KA--EKDLER LKKELEEEGA AKANLFKILG YARPEWPFIA
Hco-PGP-9.2,HCON_00130060-00001    EKT-AANKSV KA--VKDLER LKKELEEEGA VRANLIKIFR YARPEWPFIT
Hco-PGP-10,HCON_00168800-00001     --SSVGRMDI EIDVMPEYEE TEHHLPGKST NFSAVWKVIA NYREGYALLA
Hco-PGP-11,HCON_00162780-00001     --SIVSQPTT TR--P-TDKP TQIKAGRGGG IFRVYLTSL- ---RSSAVFW
Hco-PGP-13,HCON_00041390-00001     QSYNA-EADA EN--EALALE VKRIMDEDGV ISAGYIDIYK NATGNYHWIF
Hco-PGP-16,HCON_00035895-00001     --S-ISRPTE LR--GQELEN LEEEVEEKKV KGASLLDILK FARQEWCQLA
Hsa-ABCB1,NP_001335874.1           -------VRG SQ--AQDRKL STKEALDESI PPVSFWRIMK LNLTEWPYFV
Mmu-ABCB1,NP_035205.1              -------VHR KQ--DQERRL SMKEAVDEDV PLVSFWRILN LNLSEWPYLL
```

```
                                    901
Cel-PGP-1,CE11932                   IGMSTATIGG FIYPTYSVFF TSFMNVFAGN PA--DFLSQG HFWALMFLVL
Cel-PGP-2,CE41207                   GGIFGAFIFG SVTPVFALVY AEIFNVYSLP AD--QMQANV YFWCGMFVLM
Cel-PGP-3,CE03818                   ISLVFTLIRG FTWPAFSIVY GQLFKILSAG -GDD-VSIKA LLNSLWFILL
Cel-PGP-4,CE44238                   IALIFTLIRG ITWPAFSVVY GQLFKVFAEG -GED-LPVNA LISSLWFVLL
Cel-PGP-5,CE43003                   IAVLGSAIQG IYYPLSSQLM IKSYEAYAFD --KDEMLSKS HFWALSILFL
Cel-PGP-6,CE40818                   IAVFGSAIQG VSYPILAQLI VRTYEGFAMI --GEDMLYYS HLWALSFMFL
Cel-PGP-7,CE53605                   IAVFGSAIQG VSYPILAQLI VRTYEGFAMI --GEDMLYYS HLWALSFMFL
Cel-PGP-8,CE31624                   MAVIGSAIQG CNFPILSQII VQTYKAYAMD --GENILTYG HFWAAMFLVL
Cel-PGP-9,CE15714                   FAIIAALIQG AVMPAFSLFF SQIINVFSN- PDRDQMKKDG HFWALMFLVL
Cel-PGP-10,CE40807                  GAIPTTIVRA VFYLLICFQV ASVLEISIAP -DE-ERALQI FIVAAIYTAL
Cel-PGP-11,CE53819                  --LIIALLIP RAIELCSYGI GMSFAFKTLQ RSKDDYMTWN YITLAQQTLA
Cel-PGP-12,CE03260                  VGTFFAILRG SELALLANMF GFVFAAFKEP --EDEMVKAL GLIFILYVAL
Cel-PGP-13,CE53174                  IGLSAALIRG LDLPTFALLF AWVFEGFEFV PYGGKMMHRL AMSVIAHCAA
Cel-PGP-14,CE03262                  LGTVFALIRG LELPALALIF GWVFEGFTFV PYGGRMMHRM AMAVIAFASV
Hco-PGP-1,HCON_00098130-00001       VGLTACVIGG LVYPTYSVVF MQVITSF-SN PD--TLLSTG HFWALMFLVL
Hco-PGP-2,HCON_00004450-00001       LGLIACIITG TVTPTFAVLY AQIIQVYSEP VD--QMKGHV LFWCGAFIVI
Hco-PGP-3,HCON_00042800-00001       IALIFALIRG LTWPLFSIVY GKLFLLFSNP -DPNALANGN IFNSICFLLL
Hco-PGP-9.1,HCON_00130050-00001     LAVTSSIVQG CVFPAFSLFF SQIIDVFSKQ PGDPTLKSDG HFWALMFLVL
Hco-PGP-9.2,HCON_00130060-00001     IAVLSSIVQG CVFPAFSLFF TQIIDVFSKR PGDPSLRSDG HFWALMFLVL
Hco-PGP-10,HCON_00168800-00001      GAIPTTILRA FFYLLICFEV ASVLEIAIAP -AE-ERNDQI FIVAAVYTAL
Hco-PGP-11,HCON_00162780-00001      ACLIGSVLRG MELPLCAYFV GFS--YQALE QTAETFIPFM WLAIGLFIFL
Hco-PGP-13,HCON_00041390-00001      LGFVTAVFRG MELPALSLVF AYVFEAFQMV PWGADMMHRL CMAVIIFGSI
Hco-PGP-16,HCON_00035895-00001      VALILAVARG MTFPVFSIIY GQMFKTLTSG -SNSQKLHGA TMNAIWFSLL
Hsa-ABCB1,NP_001335874.1            VGVFCAIING GLQPAFAIIF SKIIGVFTRI DDPETKRQNS NLFSLLFLAL
Mmu-ABCB1,NP_035205.1               VGVLCAVING CIQPVFAIVF SRIVGVFSRD DDHETKRQNC NLFSLFFLVM

                                    951
Cel-PGP-1,CE11932                   AAAQGICSFL MTFFMGIASE SLTRDLRNKL FRNVLSQHIG FFDSPQNASG
Cel-PGP-2,CE41207                   GITFFVGFFT SANCLGRCGE SLTMKLRFEA FKNLLRQDIA FYDDLRHGTG
Cel-PGP-3,CE03818                   AFTGGISTLI SGSLLGKAGE TMSGRLRMDV FRNIMQQDAS YFDDSRHNVG
Cel-PGP-4,CE44238                   AVTSAVTTFI SGSLLGKTGE TMSSRLRMDV FKNIMQQDAT YFDDPKHNVG
Cel-PGP-5,CE43003                   AFTRPIFIFF QYYFFGKTAE KLSIKLRSMS FKHLMSLPCA FYDDPCHTAT
Cel-PGP-6,CE40818                   AVFRPLTLYC QYYYFGKVSE QLSTRLRIKS FSHMLSLPCA FYDDPNHSAT
Cel-PGP-7,CE53605                   AVFRPLTLYC QYYYFGKVSE QLSTRLRIKS FSHMLSLPCA FYDDPNHSAT
Cel-PGP-8,CE31624                   GVIRPITLYC QFFFFGKVSE KLSTRLRIKS FQHLLSLPCA FYDDPKHSPT
Cel-PGP-9,CE15714                   AAVQGTSMLF QCSLFGVAAE RLTMRIRSKV YRNVLRQDAT YFDMPKHSPG
Cel-PGP-10,CE40807                  IIVKTIFEAL GRIFIALYGH GFCKFMRNEM FRKVLRHGAA YFDEERNSPG
Cel-PGP-11,CE53819                  GITFWILHTS LMYLCGWLAN EVMNEVKQEM LSEVLNKPIP YFDNPETSPS
Cel-PGP-12,CE03260                  GLFVFITQVI SGTLFTIVAE NLGLKFRVQS FKNLLYQDAS FFDNPAHAPG
Cel-PGP-13,CE53174                  GLGIWFFQTL STVMFAIVSE NLGVRFRVAA FRNLLYQDAA YFDNPAHAPG
Cel-PGP-14,CE03262                  GVGVWFSQLA SSVLFAVVSE NLSMRFRVQS FRNLLYQDAS YFDNPAHAPG
Hco-PGP-1,HCON_00098130-00001       AGIQGCTMFG QTFFMGYGAE NLTMDLRSKL FSNILSQDMG YFDSPLHASG
Hco-PGP-2,HCON_00004450-00001       GLVHAFAFFF SAICLGRCGE ALTKKLRFEA FKNLLRQDVG FYDDIRHGTG
Hco-PGP-3,HCON_00042800-00001       GIGSGITAFA SGSLFGITGE KVAMRLRMDV FKNIMRQDAS YFDNPKHNTG
Hco-PGP-9.1,HCON_00130050-00001     GGTQAMTMLI QCFFFGLSAE RLTMRLRSKI FQNVMRMDAT YFDMPRHSAG
Hco-PGP-9.2,HCON_00130060-00001     GIIQAISMLL QCFFFGMSAE RLTMRLRSKI FNNVMRMDAT YFDMPRHSPG
Hco-PGP-10,HCON_00168800-00001      IIIKTIFEAL GRLFIALYGH GFCSCMRSIM FRKIMRHGCA YFDEERNSPG
Hco-PGP-11,HCON_00162780-00001      ALYSWLFLTV AVGFGGWSGE AATANMRVKV LKSLLSQEAE YFDRPQCSNA
Hco-PGP-13,HCON_00041390-00001      GVGVVIFQLL SSVFFAIVSE NLAMRFRVES FKNLLYQDAS YFDNPAHTPG
Hco-PGP-16,HCON_00035895-00001      GISSGISTMI SGYLFGRIGE SLTNRLRLSL FTNIVKQDGE YFDHEDHASG
Hsa-ABCB1,NP_001335874.1            GIISFITFFL QGFTFGKAGE ILTKRLRYMV FRSMLRQDVS WFDDPKNTTG
Mmu-ABCB1,NP_035205.1               GLISFVTYFF QGFTFGKAGE ILTKRVRYMV FKSMLRQDIS WFDDHKNSTG
```

```
                                         1001
Cel-PGP-1,CE11932                    KISTRLATDV PNLRTAIDFR FSTVITTLVS MVAGIGLAFF YGWQMALLII
Cel-PGP-2,CE41207                    KLCTRFATDA PNVRYVF-TR LPVVLASIVT ICGALGIGFY YGWQLALILV
Cel-PGP-3,CE03818                    SLTSRLATDA PNVQAAIDQR LAEVLTGIVS LFCGVGVAFY YGWNMAPIGL
Cel-PGP-4,CE44238                    NLTSRLATDS QNVQAAIDHR LAEVLNGVVS LFTGIAVAFW FGWSMAPIGL
Cel-PGP-5,CE43003                    RLSNRLNADS SNVTAAVDDR LGSVIMTLVA ILLAVIMSFF YSWKMSLQVL
Cel-PGP-6,CE40818                    RLSNRLNTDS SNVKAAVDDR LGCVIMTVVA ISIAITTASL YCWKMTLEVL
Cel-PGP-7,CE53605                    RLSNRLNTDS SNVKAAVDDR LGCVIMTVVA ISIAITTASL YCWKMTLEVL
Cel-PGP-8,CE31624                    RLVNRLNTDP SNIKAAVDAR LGSLLMSMVS FSLAILIACY YSWKLTLQVV
Cel-PGP-9,CE15714                    RITTRLATDA PNIKSAIDYR LGSIFNAIAS VGGGLGIAFY YGWQMAFLVM
Cel-PGP-10,CE40807                   RLVHKVINES STLNEIMEQK LDMLIPGVVC SLFSIVCALW INWKMALLCS
Cel-PGP-11,CE53819                   ACVSRIISHA HNCYACLDHR AIRFVWFIAG TIFSLLLAFP FVWELGVLGL
Cel-PGP-12,CE03260                   KLITRLASDA PNVKAVVDTR MLQVIYSITS ITINLITGYI FCWRIAIAGT
Cel-PGP-13,CE53174                   SLITRLAADP PCVKAVVDGR MMQVVYATAA VIACVTIGFI NCWQVAILGT
Cel-PGP-14,CE03262                   KLITRLASDA PNIKAVVDAR MLQVIYALAA IIANIAIAFI YCWQIGILGT
Hco-PGP-1,HCON_00098130-00001        KICTRLATDV PNLRSAIDFR LSTVIMTLIS MLAGIVLAFF YGWQMAFLVV
Hco-PGP-2,HCON_00004450-00001        KLCTRFATDA PNVRYVF-TR LPGVLSSVVT IIGALVIGFI FGWQLALILM
Hco-PGP-3,HCON_00042800-00001        NLTAHLASDT PNVQAAIDQR LAEVLQGVCA LVAGIAVAFS FGWNVAPIGL
Hco-PGP-9.1,HCON_00130050-00001      KITTRLATDA PNVKSALDYR FGSVFSSVVS ICCGIGIAFY FGWQMALLTI
Hco-PGP-9.2,HCON_00130060-00001      KITTRLATDA PNVKSALDYR FGAVFSSLVS VGCGIGIAFY SGWQMALLAI
Hco-PGP-10,HCON_00168800-00001       RILQRIITDS STLNKIMESK LDILIPAVIC PLFSLAAAMY INWKMALLCS
Hco-PGP-11,HCON_00162780-00001       ACVAELSTKA PDVQACLDYR FMLMVNNMCA VVVCIPLSII ACWESGVA--
Hco-PGP-13,HCON_00041390-00001       KLITRLASDA PNIKAVVDGR ALQVIYAMTA VIACIIIGFI SSWQVTLMGI
Hco-PGP-16,HCON_00035895-00001       KLTTRLATDA PNIRAAIDQR LADVVSAVSS IIGGISIAFS YGPAMAPIGV
Hsa-ABCB1,NP_001335874.1             ALTTRLANDA AQVKGAIGSR LAVITQNIAN LGTGIIISFI YGWQLTLLLL
Mmu-ABCB1,NP_035205.1                SLTTRLASDA SSVKGAMGAR LAVVTQNVAN LGTGVILSLV YGWQLTLLLV

                                         1051
Cel-PGP-1,CE11932                    AILPIVAFGQ ---YLRGRRF TGKNVKSASE FADSGKIAIE AIENVRTVQA
Cel-PGP-2,CE41207                    VMVPLLVMGG ---YFEMQMR FGKQIRDTQL LEEAGKVASQ AVEHIRTVHS
Cel-PGP-3,CE03818                    ATALLLVVVQ ---SSVAQYL KFRGQRDMDS AIEASRLVTE SISNWKTVQA
Cel-PGP-4,CE44238                    ITALLLVIAQ ---SAVAQYL KYRGPKDMES AIEASRIVTE SISNWKTVQA
Cel-PGP-5,CE43003                    MFCPLLYLAG ---YCNDNFV DQAVEEDSIA FEKSNRAAIE ALENVRTVRA
Cel-PGP-6,CE40818                    MFFPLLYLAE ---YCYEAAT ETAIQEDTIA FENSNRTAIE ALENVRTVRA
Cel-PGP-7,CE53605                    IFFPLLYLAE ---YCYDAAT ETSIQEDTIA FENSNRTAIE ALENMRTVRA
Cel-PGP-8,CE31624                    LFFPVLYYAK ---FLYKKTT TISIKEDSVA FENSNKIAIE VLDNMKTVKA
Cel-PGP-9,CE15714                    AIFPFMAVGQ ---ALMMKYH GGSATSDAKE MENAGKTAME AIENIRTVQA
Cel-PGP-10,CE40807                   FQFPAYFVIR ---ILQIREG TKRQRQMVDE EKKAANLASV VLANMSTIKA
Cel-PGP-11,CE53819                   GITILLTIFS LHFVSVA--- -HRAHSEKSV MDKSGEFAVE IVEHIRAIKL
Cel-PGP-12,CE03260                   IMIVLFATMM ---ISMAYKI AR-ENLKQIR KDEAGKIAIE IIESVKTIQL
Cel-PGP-13,CE53174                   ALIFLLGFIM ---AGLAFKI SI-VAAEHME NDDAGKIAIE IIENVKTIQL
Cel-PGP-14,CE03262                   SLILLLAFVM ---IGLAYKI SL-MNVEQIQ NDDAGRIAIE IIENVKTIQL
Hco-PGP-1,HCON_00098130-00001        GILPLLGIGQ ---ALRVRVM GGKHRKNAKD FEDSGKVAME AIEHVRTVQA
Hco-PGP-2,HCON_00004450-00001        VMVPLIIGSG ---YFEMRMQ FGKKMRDTEL LEEAGKVASQ AVENIRTVHA
Hco-PGP-3,HCON_00042800-00001        ATALMLVVIQ ---SSVAQYL KFRGQKDMDS AVEASQVATE SISNTRTIQA
Hco-PGP-9.1,HCON_00130050-00001      AIFPLAAVGQ ---AIQMRFM SGRATADAKE MENSGKIAME AIENIRTVQA
Hco-PGP-9.2,HCON_00130060-00001      SIFPLGAAGR ---AIQMRFM SGRATADAKE MENSGKIAME AIEHIRTVQA
Hco-PGP-10,HCON_00168800-00001       FQFPAYFVIR ---IVQMKEG TKRQRAMVEE ENKAANLASV VLSNMGTIKA
Hco-PGP-11,HCON_00162780-00001       -MTVLLALFI VSMWITSNR- ISASMEKKSE IDKTPELSIE VFEHAKTIQL
Hco-PGP-13,HCON_00041390-00001       GMLIVLATSM ---IWLALTI MN-KNIELVK DDEAGRIAIE TIENVRTIQL
Hco-PGP-16,HCON_00035895-00001       LTAVTLITLQ ---TLVARYL KVRGQRDAVL AEEPSRLATE AIEQHKTVQY
Hsa-ABCB1,NP_001335874.1             AIVPIIAIAG ---VVEMKML SGQALKDKKE LEGSGKIATE AIENFRTVVS
Mmu-ABCB1,NP_035205.1                VIIPLIVLGG ---IIEMKLL SGQALKDKKQ LEISGKIATE AIENFRTIVS
```

	1101				
Cel-PGP-1,CE11932	LAREDTFYEN	FCEKLDIPHK	EAIKEAFIQG	LSYGCASSVL	YLLNTCAYRM
Cel-PGP-2,CE41207	LNRQEQFHFT	YCEYLREPFN	TNLKHAHTYG	AVFAFSQSLI	FFMYAAAFYL
Cel-PGP-3,CE03818	LTKQEYMYDA	FTAASKSPHR	RAIVRGLWQS	LSFALAGSFV	MWNFAIAYMF
Cel-PGP-4,CE44238	LTKQEYMFHA	FTAASKNPRK	RAFTKGLWQS	LSFALAGSFF	LWNFAIAYMF
Cel-PGP-5,CE43003	LNMENRVILL	VTSHLQKIRN	SYFKRAVIQG	TANGFACSCY	FFIYAVSFKF
Cel-PGP-6,CE40818	LNLEDKIMSL	ISEHLQKIHK	SYFKRAIIQG	AANGLSLSCF	LFVYSVSFKF
Cel-PGP-7,CE53605	LNLEDKIMSL	ISEHLQKIHK	SYFKRAIIQG	AANGLSLSCY	LFVYSVSFKF
Cel-PGP-8,CE31624	LNMEARVINL	VMEYLAVLKT	SYPRRSVVMG	LANGFSAGCS	QIVYAISFKF
Cel-PGP-9,CE15714	LTLQTKLYNI	FCSHLDAPHG	GNISKAIIRG	LTYGFANSIQ	FFTYAAAFRF
Cel-PGP-10,CE40807	YNLQSHFYNV	FCDALKPVSQ	CMQKQSLISA	LVFACQYSFT	YILIAITLHF
Cel-PGP-11,CE53819	LAVEGYFENK	FSEYLKTSEI	YENKIGFVSS	LNFAITQSYV	FACDMLLFFV
Cel-PGP-12,CE03260	LTSTQRFLTH	YKDAQLVQHK	SEMRKSYIQS	VNNAISQTFM	YFAMFVCYGV
Cel-PGP-13,CE53174	LTRTRRFLNS	YENESKKRKR	TELRKSVYEA	VNYCISQNFM	YYMSCFCFAL
Cel-PGP-14,CE03262	LTRCELFFDH	YQTSSKQQKR	SELKKGMIEA	INYSLTQSFM	YFMMCFTYAV
Hco-PGP-1,HCON_00098130-00001	LTKEEAFHQK	FCDYLDAPHR	DALRESFIQG	VAYGFATSIV	YVLNCCSYRL
Hco-PGP-2,HCON_00004450-00001	LNRQEQFHFM	YCEYLKEPYR	ENLCQAHTYG	GVFAFSQSLL	FFMYAVAFWI
Hco-PGP-3,HCON_00042800-00001	LCKEGYMYEA	YCAAAQEPHR	RALVRGLWQA	LSLALSNSFV	VVNFAIAYAF
Hco-PGP-9.1,HCON_00130050-00001	LTLERRLHAQ	FCHHLDGPHK	TSRRKALIQG	VSYGFASSIF	YFLYASCFRF
Hco-PGP-9.2,HCON_00130060-00001	LTLERRLHAE	FCHHLDGPHK	TNRRKALIQG	VSYGFACSIF	YFLYASCFRF
Hco-PGP-10,HCON_00168800-00001	YNLQEHFYSV	FANTLEPLAR	AMKRQSVISA	FVFACQYSFT	YILIALTLYF
Hco-PGP-11,HCON_00162780-00001	LAVQDYFLQK	YESYEAVVKK	QEKWTTIYQS	IQFGLTQSYI	YFSDLVTYGI
Hco-PGP-13,HCON_00041390-00001	LTRMSTFYGR	YKAASKLGKR	SESIKGIFEA	INFTISQSFT	YLMVCVCYAV
Hco-PGP-16,HCON_00035895-00001	LTKERQFLDK	FVTQMHGPHK	RAIFRGIVQS	LTYALSVSFV	NLNFAIAYLY
Hsa-ABCB1,NP_001335874.1	LTQEQKFEHM	YAQSLQVPYR	NSLRKAHIFG	ITFSFTQAMM	YFSYAGCFRF
Mmu-ABCB1,NP_035205.1	LTREQKFETM	YAQSLQVPYR	NAMKKAHVFG	ITFSFTQAMM	YFSYAACFRF

	1151				
Cel-PGP-1,CE11932	GLALIITDPP	TMQPMRVLRV	MYAITISTST	LGFATSYFPE	YAKATFAGGI
Cel-PGP-2,CE41207	GSIFVNQQ--	AMQPIDVYRV	FFAISFCGQM	IGNTTSFIPD	VVKARLAASL
Cel-PGP-3,CE03818	GLWLISNN--	WSTPYTVFQV	IEALNMASMS	VMLAASYFPE	YVRARISAGI
Cel-PGP-4,CE44238	GLWLISNN--	WTTPFAVFQV	IEALNMASMS	VMMAASYFPE	YVRARISAGI
Cel-PGP-5,CE43003	GTWLVLRE--	EILPMDTYLV	LMTLSMTASY	AGSAVAYLPD	HRKAIHAAGL
Cel-PGP-6,CE40818	GTYLALRK--	EVAPMDTYLV	LMTLSMTANM	AGSAAAYLPD	YKKAVHAAGL
Cel-PGP-7,CE53605	GTYLALRK--	EVAPMDTYLI	LETLSMTANM	AGSAAAYLPD	YKKAVHAAGL
Cel-PGP-8,CE31624	GTYLILQK--	EVIPMDMYLS	LITLSYTSNM	AGSAISYMPD	FRKAIHSAGL
Cel-PGP-9,CE15714	GLFLIFDKNV	LMEPENVLRV	LFAISFSFGT	IGFAASYFPE	YIKATFAAGL
Cel-PGP-10,CE40807	GKVMMLAN--	EISVFDYMRV	VLLTQFGANF	FSQLIASVSD	FTKAQIAAEN
Cel-PGP-11,CE53819	GTLLIYHG--	RYSPDKVFLA	FNGAQMSAWG	VMYFSPWFPE	IVRGSASANQ
Cel-PGP-12,CE03260	GTPLMYHG--	IVEAEPTFRA	INCMMMGSVA	VMHSSHNFPE	FVKAKTAAGM
Cel-PGP-13,CE53174	AIRIINQG--	DQTVDKTFRC	LMAMMLCCEG	IIMSAQFFPQ	FVGAKSAAGQ
Cel-PGP-14,CE03262	GIRIIYQG--	DKSSDDTFKG	IIAMMLGAVA	VMNSAQYFPE	FVKAKTAAGM
Hco-PGP-1,HCON_00098130-00001	GLYLIVS--S	IMMPTRVLRV	MYAITISSST	LGFASAYFPE	YMKAAFAGGI
Hco-PGP-2,HCON_00004450-00001	GAIFVDNH--	SMQPIDVYRV	FFAFMFCGQM	VGNISSFIPD	VVKARLAASL
Hco-PGP-3,HCON_00042800-00001	GLWLIRNG--	WSTPFIVFQV	IEALNMASMT	VMMAASYFPE	YIRARISAGV
Hco-PGP-9.1,HCON_00130050-00001	GLWLIVNG--	TIHSMNVLRV	LFAISFTAGS	MGFASSYFPE	YIKATFAAGI
Hco-PGP-9.2,HCON_00130060-00001	GAWLIVHG--	YLGPMNVLRV	ELAISFTAGS	LGFASSYFPE	YIKATFAAGL
Hco-PGP-10,HCON_00168800-00001	GKGMMLNN--	EITVFDYMRV	VLLTQFGANF	FSQLVASVSD	LSKARVAAEN
Hco-PGP-11,HCON_00162780-00001	GASMIYFG--	RVDSKDTVVS	ATSANFAGWA	VIFASAALGD	FVRSHFAAQS
Hco-PGP-13,HCON_00041390-00001	GIHIIYTE--	QKTPDNVFRT	IIAMLLASVA	VMNSSSYFPE	FVKARTAAGL
Hco-PGP-16,HCON_00035895-00001	GIWLVGRR--	ICSPYTVFQV	IESLNTASMS	LIAFATYFPE	YVRARLSAAL
Hsa-ABCB1,NP_001335874.1	GAYLVAHK--	LMSFEDVLLV	FSAVVFGAMA	VGQVSSFAPD	YAKAKISAAH
Mmu-ABCB1,NP_035205.1	GAYLVAQQ--	LMTFENVMLV	FSAVVFGAMA	AGNTSSFAPD	YAKAKVSASH

```
                                 1201
Cel-PGP-1,CE11932                   IFGMLRKIS- KIDSLSLAGE KKK-LYGKVI FKNVRFAYPE RPEIEILKGL
Cel-PGP-2,CE41207                   LFYLIEHPT- PIDSLSDSGI VKP-ITGNIS IRNVFFNYPT RKDTKVLQGF
Cel-PGP-3,CE03818                   MFTMIRQKS- VIDNRGLTGD TPT-IKGNIN MRGVYFAYPN RRRQLVLDGF
Cel-PGP-4,CE44238                   MFTMIRQKA- KIDNRGLTGE TPD-IRGDIS MKGVYFAYPN RNRQLILNNF
Cel-PGP-5,CE43003                   IFHLFTYPA- IMPYDSSQGK RNI-KNGEIE LKNVSFEYAQ RSDKMILDGV
Cel-PGP-6,CE40818                   IFHLFTYPA- TMPFSSSDGK KNI-EKGEII GENVQFHYDQ RPDRMILNGV
Cel-PGP-7,CE53605                   IFHLFTYPA- TMPFSSSDGK KNI-EKGEII GENVQFHYDQ RPDRMILNGV
Cel-PGP-8,CE31624                   IFNLFTYPA- TMPFNSDTGS RSI-TKGEVN GENVKFHYHQ RPDYTVLDSV
Cel-PGP-9,CE15714                   IFNMLEEEP- RIDGMTSSGT YPQ-LSGEVK LNKVFFRYPE RPAVPILQGL
Cel-PGP-10,CE40807                  VMRVIREPPV DMDNLSEEGL RPK-LEGNLC LKDVSFRYPS RPIVPVLTNL
Cel-PGP-11,CE53819                  IFSFFDKNR- T--NSTSGLS KPE-INGKVE VSDVTFAYPS TPHRNVCEGF
Cel-PGP-12,CE03260                  LFKLIYRKS- KTGDV-MEGN NTE-IRGNVL FESVKFSYPQ RPMQPVMTDL
Cel-PGP-13,CE53174                  MFNLINRQP- QTGDL-KSGT KPE-IRGNIL FENVKFSYPQ RPHQPVMKQL
Cel-PGP-14,CE03262                  LFNIIYRKP- RTGDL-MEGD RPE-IRGNIL FENVKFSYPQ RPLQPIMKGL
Hco-PGP-1,HCON_00098130-00001       IFNMLKQKS- SIDNLTHDGK KEK-LSGAIT FKNVKFSYPE RPQIEVLKGL
Hco-PGP-2,HCON_00004450-00001       LFYLIEHPS- EIDNLSEDGV TKK-ISGHIS FRNVYFNYPT RRQIRVLRGL
Hco-PGP-3,HCON_00042800-00001       MFTMMRQRP- KIDNMSHQGE KPA-LKGDVA LRNVYFSYPA RRRQLVLQGM
Hco-PGP-9.1,HCON_00130050-00001     IFHMLEEEP- RIDGMTNNGK KPK-ITGAVK LNKVYFKYPE RPDVPILQGL
Hco-PGP-9.2,HCON_00130060-00001     IFHMLEEKP- RIDGMSSNGK KPK-ILGAVK LDQVYFNYPE RPDVPILQGL
Hco-PGP-10,HCON_00168800-00001      VLSVLKEGAV DFDNLSEEGQ RPK-LEGTIK FKDVSFRYPT RPVVPILDKL
Hco-PGP-11,HCON_00162780-00001      LYALIDSYK- ----KAEGGA TPE-LDGSIK VEKVTFSYPS RPDVKVAQNL
Hco-PGP-13,HCON_00041390-00001      LFSVIYRKP- RTGDA-NVGD KVT-IRGNIL FDDVKFSYPQ RPRQPIMRGL
Hco-PGP-16,HCON_00035895-00001      LFRMLRDKP- KIDSLSPLGM QTK-LRGSIH FSDLSFSYPV SRRDMVLKGI
Hsa-ABCB1,NP_001335874.1            IIMIIEKTP- LIDSYSTEGL MPNTLEGNVT FGEVVFNYPT RPDIPVLQGL
Mmu-ABCB1,NP_035205.1               IIRIIEKTP- EIDSYSTEGL KPTLLEGNVK FNGVQFNYPT RPNIPVLQGL

                                 1251
Cel-PGP-1,CE11932                   SFSVEPGQTL ALVGPSGCGK STVVALLERF YDTLGGEIFI DGSEIKTLNP
Cel-PGP-2,CE41207                   TLDIKAGKTV ALVGHSGCGK STIMGLLERF YNQDKGMIMI DGDNIRNLNI
Cel-PGP-3,CE03818                   NMSANFGQTV ALVGPSGCGK STTIQLIERY YDALCGSVKI DDSDIRDLSV
Cel-PGP-4,CE44238                   NMSAQFGETV ALVGPSGCGK STSIQLIERY YDAICGAVKI DDHDIRDISV
Cel-PGP-5,CE43003                   SLKLPAGRTL ALVGPSGSGK STIISLLERF YHAVDGEVKI DEENVVDVNL
Cel-PGP-6,CE40818                   NLKVDPGKTL ALVGPSGCGK STIISLLERF YHAVDGEVKI DSENVEDINL
Cel-PGP-7,CE53605                   NLKVDPGKTL ALVGPSGCGK STIISLLERF YHAVDGEVKI DSENVEDINL
Cel-PGP-8,CE31624                   NLKVEAGKTL AIVGPSGSGK STIISLLEMF YRADQGFIKI DNDNVENINL
Cel-PGP-9,CE15714                   NVHVKPGQTL ALVGPSGCGK STVISLLERL YDPLEGAVTV DNNDLRQMNP
Cel-PGP-10,CE40807                  NLKVRGGESI ALVGPSGSGK SSVISLFQRM YNATDGVVII DKYNIKSINP
Cel-PGP-11,CE53819                  SLNIPKGHSI ALVGASGCGK STIISMLERF YSAKAGRISV DDNDIDGIDV
Cel-PGP-12,CE03260                  HFSAHSGQTV ALVGPSGTGK STCIAMLERF YDVSGGALRI DGQNIKSLSL
Cel-PGP-13,CE53174                  QWTALRGQTV ALVGPSGSGK STCIGMLERF YDVTGGALRM DGQDIKNISL
Cel-PGP-14,CE03262                  QWTALRGQTV ALVGPSGSGK STNIGMLERF YDVTGGALRI DGQDIRKLSL
Hco-PGP-1,HCON_00098130-00001       SFTAKPGETL ALVGPSGCGK STVVSLIERF YDVKAGQVLL DSHDLRSLNP
Hco-PGP-2,HCON_00004450-00001       NLEINPGTTV ALVGQSGCGK STVMALLERF YNQNKGVITV DGENIRNMNI
Hco-PGP-3,HCON_00042800-00001       NLSVRHGQTV ALVGASGCGK STVIQLVERY YDALCGTVSI DTYDIRDLSI
Hco-PGP-9.1,HCON_00130050-00001     DINVKPGETL ALVGPSGCGK STVISLLERL YDALDGSVEI DGNDLREVNP
Hco-PGP-9.2,HCON_00130060-00001     NIRVEPGETL ALVGPSGCGK STVISLLERF YDALDGSVEI DGNDIREMNP
Hco-PGP-10,HCON_00168800-00001      NLEIRAGQSV ALVGPSGSGK TSVMALIQRL YNATDGEVLM DKYNVRSINP
Hco-PGP-11,HCON_00162780-00001      NLMARCGQAI ALVGASGCGK STVIQLLERF YEPDSGNIKI DNHELKQLCR
Hco-PGP-13,HCON_00041390-00001      QFSAQRGQTV ALVGPSGSGK STIISMLERF YDTTGGYVRF DGKDIKTLSL
Hco-PGP-16,HCON_00035895-00001      TLKVPAGKTV ALVGPSGCGK STSIQLIERF YDPVAGSVLF DEVDARELNL
Hsa-ABCB1,NP_001335874.1            SLEVKKGQTL ALVGSSGCGK STVVQLLERF YDPLAGKVLL DGKEIKRLNV
Mmu-ABCB1,NP_035205.1               SLEVKKGQTL ALVGSSGCGK STVVQLLERF YDPMAGSVFL DGKEIKQLNV
```

```
                                                   1301
Cel-PGP-1,CE11932                  EHTRSQIAIV SQEPTLFDCS IAENIIYGLD PSSVTMAQVE EAARLANIHN
Cel-PGP-2,CE41207                  SSLREQVCIV SQEPTLFDCT IGENICYGTN R-NVTYQEIV EAAKMANIHN
Cel-PGP-3,CE03818                  KHLRDNIALV GQEPTLFNLT IRENITYGLE N--ITQDQVE KAATLANIHT
Cel-PGP-4,CE44238                  KHLRHNIALV GQEPTLFNLT IRENITYGLE N--VSQEQVE KAATLANIHS
Cel-PGP-5,CE43003                  HHLRESVSLV SQEPVLFNCS IKENFLFGIS H-NASQLEID QALKVANAFS
Cel-PGP-6,CE40818                  NHLRSNLALV SQEPTLFNCS IRENLLYGLT R-SVPQLELE KALQTANAFN
Cel-PGP-7,CE53605                  NHLRSNLALV SQEPTLFNCS IRENLLYGLT R-SVPQLELE KALQTANAFN
Cel-PGP-8,CE31624                  DHLRSNLGLV SQGPVLFNCS IRDNILYGLT R-NISQTEIE NALQIANAFN
Cel-PGP-9,CE15714                  KHLRKHIALV SQEPILFDTS IRENIVYGLQ PGEYTHEQIE TACSKANIHK
Cel-PGP-10,CE40807                 AYLRRCIVQV GQEPDLFSFT IKENIAFGMM ESEATIDKVI EAAKIADIHN
Cel-PGP-11,CE53819                 NHLRNNISVV GQEPVLFNAT IRENITIGID E--VSVEEVQ KACKAANAAG
Cel-PGP-12,CE03260                 HHLRTQMALV GQEPRLFAGT IKENVCFGLK D--VSVEKVH QALELANASR
Cel-PGP-13,CE53174                 YHLRTQMALV GQEPRLFVGT IRENVCLGLK D--VPLEKIN QALELANANR
Cel-PGP-14,CE03262                 FHLRTQMALV GQEPRLFAGT IRENVCLGLK D--VPLEKIN QALELANANR
Hco-PGP-1,HCON_00098130-00001      YHTRSQIAIV SQEPILFDCS IADNIVYGME VRPS-QTEIE NAARKANIHT
Hco-PGP-2,HCON_00004450-00001      RNLREQVCIV SQEPTLFDCT IMENICYGLD DPKPSYEQVV AAAKMANIHN
Hco-PGP-3,HCON_00042800-00001      RYVRDNMALV GQEPTLFNVT IRENIMYGLD K--CSQEEIV HAARLANIHD
Hco-PGP-9.1,HCON_00130050-00001    THLRAHIALV SQEPILFDRS IRDNILYGLP PGSVSDAAVH EVAQRANIHK
Hco-PGP-9.2,HCON_00130060-00001    SHLRAHIALV SQEPILFDRS IRDNILYGLP PGSVSEAEVH EIAQRANIHK
Hco-PGP-10,HCON_00168800-00001     AYLRRMVVSV GQEPTLFSFT IKENIAYGMM ESEVTMEKIQ EAAKIANIHD
Hco-PGP-11,HCON_00162780-00001     VHLRNNIALV GQEPILFKGS IIENITLGLE D--VSVAEVQ EACRQANAAN
Hco-PGP-13,HCON_00041390-00001     NHLRTQMALV GQEPRLFSGT IKQNICFGLG V--VPMEKID RALELANAKG
Hco-PGP-16,HCON_00035895-00001     RHLRSQMSLV GQEPILFNYS IRENIAYGLE Q--ATVDQIE NAAKLANAHN
Hsa-ABCB1,NP_001335874.1           QWLRAHLGIV SQEPILFDCS IAENIAYGDN SRVVSQEEIV RAAKEANIHA
Mmu-ABCB1,NP_035205.1              QWLRAHLGIV SQEPILFDCS IAENIAYGDN SRAVSHEEIV RAAKEANIHQ

                                                   1351
Cel-PGP-1,CE11932                  FIAELPEGFE TRVGDRGTQL SGGQKQRIAI ARALVRNPKI LLLDEATSAL
Cel-PGP-2,CE41207                  FILGLPDGYD THVGEKGTQL SGGQKQRIAI ARALVRSPSV LLLDEATSAL
Cel-PGP-3,CE03818                  FVMGLPDGYD TSVGASGGRL SGGQKQRVAI ARAIVRDPKI LLLDEATSAL
Cel-PGP-4,CE44238                  FVENLPEGYD TSVGASGGRL SGGQKQRIAI ARAIVRNPKI LLLDEATSAL
Cel-PGP-5,CE43003                  FVSQFPQGLD TLVGERGAQL SGGQKQRIAI ARAILRNPKV LLLDEATSAL
Cel-PGP-6,CE40818                  FVFQFPQGLD TLVGERGAQL SGGQKQRIAI ARAILRNPKV LLLDEATSAL
Cel-PGP-7,CE53605                  FVFQFPQASF NCAQLT---- ---------- ---------- ----------
Cel-PGP-8,CE31624                  FVFQLPQGLD TIVGDRGAQL SGGQKQRIAI TRAILRNPKL LLLDEATSAL
Cel-PGP-9,CE15714                  FIDELPDGYE TRVGEKGTQL SGGQKQRIAI ARALIRNPKI LLLDEATSAL
Cel-PGP-10,CE40807                 FITSLPQGYD TEVGEFGAQL SGGQKQRIAI ARAIIRKPTV LLLDEATSAL
Cel-PGP-11,CE53819                 FIESFPLGYD TIVGEGGASL SGGQKQRIAI ARAIIRKPKI LLLDEATSAL
Cel-PGP-12,CE03260                 FLANLPAGID TEVGEKGGQL SGGQKQRIAI ARALVRDPKI LLLDEATSAL
Cel-PGP-13,CE53174                 FLGNLPDGID TEVGERGGQL SGGQKQRIAI ARALVRDPKI LLLDEATSAL
Cel-PGP-14,CE03262                 FLANLPAGID TDVGEKGGQL SGGQKQRIAI ARALVRDPKI LLLDEATSAL
Hco-PGP-1,HCON_00098130-00001      FIKGLPDGYE TGVGDKGTQL SGGQKQRIAI ARALIRSPKI LLLDEATSAL
Hco-PGP-2,HCON_00004450-00001      FVLGLPEGYD TRVGEKGTQL SGGQKQRIAI ARALIRDPPI LLLDEATSAL
Hco-PGP-3,HCON_00042800-00001      FIASLPEEYN TVVGAKGGLL SGGQKQRIAI ARAIVRDPKI LLLDEATSAL
Hco-PGP-9.1,HCON_00130050-00001    FIMDLPDGYN TRAGEKGTQL SGGQKQRIAI ARALIRNPKI LLLDEATSAL
Hco-PGP-9.2,HCON_00130060-00001    FVMELPEGYN TRAGEKGVQL SGGQKQRIAI ARALIRNPKI LLLDEATSAL
Hco-PGP-10,HCON_00168800-00001     FIMSLPQGYD TEIGEFGAQL SGGQKQRIAI ARAIVRRPVV LLLDEATAAL
Hco-PGP-11,HCON_00162780-00001     FVEAFPQGYE TDVGEKGGSL SGGQKQRIAI ARALIRKPKV ILLDEATSAL
Hco-PGP-13,HCON_00041390-00001     FLANLPAGID TEVGEKGTQL SGGQKQRIAI ARALVRDPKI LLLDEATSAL
Hco-PGP-16,HCON_00035895-00001     FIIKLPSGYD TIVGERGSML SGGQKQRIAI ARAVIRDPKI LLLDEATSAL
Hsa-ABCB1,NP_001335874.1           FIESLPNKYS TKVGDKGTQL SGGQKQRIAI ARALVRQPHI LLLDEATSAL
Mmu-ABCB1,NP_035205.1              FIDSLPDKYN TRVGDKGTQL SGGQKQRIAI ARALVRQPHI LLLDEATSAL
```

```
                                    1401
Cel-PGP-1,CE11932                   DTESEKVVQE ALDRA----R EGRTCIVIAH RLNTVMNADC IAVVSNGTII
Cel-PGP-2,CE41207                   DTESEKIVQE ALDAA----K QGRTCLVIAH RLSTIQNSDV IAIVSEGKIV
Cel-PGP-3,CE03818                   DTESEKIVQE ALDKA----R LGRTCVVIAH RLSTIQNADK IIVCRNGKAI
Cel-PGP-4,CE44238                   DTESEKIVQE ALDKA----R LGRTCVVIAH RLSTIQNADK IIVCRNGKAI
Cel-PGP-5,CE43003                   DSDSEKVVQN ALDTA----S ERLSTVVVAH RLSTVVNADS IAVLKNGKVA
Cel-PGP-6,CE40818                   DSDSEKVVQN ALDTA----S ERLSTVVVAH RLSTVVNADS IAVLKNGKVA
Cel-PGP-7,CE53605                   ---------- ---------- ---------- ---------- ----------
Cel-PGP-8,CE31624                   DTESEKIVQN ALDTA----S ERLSTIVVAH RLSTIINADS IAVLRNGKVV
Cel-PGP-9,CE15714                   DTESEKQVQV ALDAA----A KDRTCIVVAH RLSTIVNAGC IMVVKNGQVV
Cel-PGP-10,CE40807                  DSASEREVQN AFERVKRSTK TQCTCIQIAH RLSTIRNVDK IYVIVHGEIA
Cel-PGP-11,CE53819                  DTQSEEIVQK ALRSA----T TGRTSIIVAH RLSTVQHCDT IYYISRGAVA
Cel-PGP-12,CE03260                  DSESERAVQE ALDRA----R EGRTCITIAH RLSSIQNSDL IVYIDHGMVQ
Cel-PGP-13,CE53174                  DSESERAVQE ALDRA----R EGRTCITIAH RLSSIQNSDL IVYIDDGRVQ
Cel-PGP-14,CE03262                  DSESERAVQE ALDRA----R EGRTCITIAH RLSSIQNSDL IVYIDKGKVQ
Hco-PGP-1,HCON_00098130-00001       DTESEKVVQE ALDRA----R EGRTCIVIAH RLSTVVNADC IAVVKGGLII
Hco-PGP-2,HCON_00004450-00001       DTESEKIVQD ALEVA----R QGRTCLVIAH RLSTIQDSDV IVMIQEGKAT
Hco-PGP-3,HCON_00042800-00001       DTESEKVVQE ALDRA----R LGRTCLVIAH RLSTIQNADH IVVCRDGRVA
Hco-PGP-9.1,HCON_00130050-00001     DTESEKVVQE ALDKA----S EGRTCIVVAH RLSTVVNANC IMVVKGGKVV
Hco-PGP-9.2,HCON_00130060-00001     DTESEKVVQE ALDKA----S EGRTCIVVAH RLSTVVNANC IMVVQGGKIV
Hco-PGP-10,HCON_00168800-00001      DSTSEKAVQL ALEKA----Q KSCTCLQIAH RLSSIRGVDK ICVLVDGAIV
Hco-PGP-11,HCON_00162780-00001      DTESEKVVQN ALNEA----S HGRTSITIAH RLSTVRDADR IYYIENGAVV
Hco-PGP-13,HCON_00041390-00001      DSESERAVQK ALDLA----R EGRTCITIAH RLSSIQNADL IVYVENGKVR
Hco-PGP-16,HCON_00035895-00001      DTESEKIVQE ALEKA----R QGRTCIVIAH RLSSIQNADL IIVIKDGMVE
Hsa-ABCB1,NP_001335874.1            DTESEKVVQE ALDKA----R EGRTCIVIAH RLSTIQNADL IVVFQNGRVK
Mmu-ABCB1,NP_035205.1               DTESEKVVQE ALDKA----R EGRTCIVIAH RLSTIQNADL IVVIENGKVK

                                    1451
Cel-PGP-1,CE11932                   EKGTHTQLMS EKGAYYKLTQ KQMTEKK--- ---------- -------
Cel-PGP-2,CE41207                   EKGTHDELIR KSEIYQKFCE TQRIVESQ-- ---------- -------
Cel-PGP-3,CE03818                   EEGTHQTLLA RRGLYYRLVE KQSS------ ---------- -------
Cel-PGP-4,CE44238                   EEGTHQTLLA RRGLYYRLVE KQST------ ---------- -------
Cel-PGP-5,CE43003                   EQGTHEELLR KRSIYWRLVQ KQGIQVETLI E--------- -------
Cel-PGP-6,CE40818                   EQGTHEELLR KRSIYWRLVQ KQGIQVETPS D--------- -------
Cel-PGP-7,CE53605                   ---------- ---------- ---------- ---------- -------
Cel-PGP-8,CE31624                   EQGTHNQLLA VKGDYWRLVQ HQKSS----- ---------- -------
Cel-PGP-9,CE15714                   EQGTHNELIA KRGAYFALTQ KQSSNQSGGA FDTSEALDDD DDDHVKF
Cel-PGP-10,CE40807                  EEGSHEQLIN LKGIYYEMNQ MT-------- ---------- -------
Cel-PGP-11,CE53819                  EYGTHAELVA MDSKYARLVA AQSLS----- ---------- -------
Cel-PGP-12,CE03260                  EAGNHAHLMS LRGKYYNLIK KQDLTT---- ---------- -------
Cel-PGP-13,CE53174                  ESGTHKELMQ LKGKYFELIK KQDLAI---- ---------- -------
Cel-PGP-14,CE03262                  EAGNHTQLMH QKGRYYKLIK KQDLAV---- ---------- -------
Hco-PGP-1,HCON_00098130-00001       EQGTHTELMA KRGFYYELTQ KQTVKSASE- ---------- -------
Hco-PGP-2,HCON_00004450-00001       DRGTHEHLLM KNDLYKRLCE TQRLVESQ-- ---------- -------
Hco-PGP-3,HCON_00042800-00001       EHGTHQSLLS RKGIYYKLVE RQNR------ ---------- -------
Hco-PGP-9.1,HCON_00130050-00001     EKGTHSELMQ AKGAYWALTQ KQTLAKG--- ---------- -------
Hco-PGP-9.2,HCON_00130060-00001     EKGTHNELMQ AKGVYWELTQ KQTTAKE--- ---------- -------
Hco-PGP-10,HCON_00168800-00001      EEGTHDALIA KRGLYYSMTQ SA-------- ---------- -------
Hco-PGP-11,HCON_00162780-00001      EYGTHEELIE ADGKYALLVK AQQLAKTD-- ---------- -------
Hco-PGP-13,HCON_00041390-00001      ESGTHSQLMQ RRGCYYQLIK KQDLTT---- ---------- -------
Hco-PGP-16,HCON_00035895-00001      EQGTHQQLLA REGLYASMVT KQDLK----- ---------- -------
Hsa-ABCB1,NP_001335874.1            EHGTHQQLLA QKGIYFSMVS VQAGTKRQ-- ---------- -------
Mmu-ABCB1,NP_035205.1               EHGTHQQLLA QKGIYFSMVQ AGAK--RS-- ---------- -------
```
